# Synthesis, Computational Study, and In Vitro α-Glucosidase Inhibitory Action of Thiourea Derivatives Based on 3-Aminopyridin-2(1*H*)-Ones

**DOI:** 10.3390/molecules29153627

**Published:** 2024-07-31

**Authors:** Zarina Shulgau, Irina Palamarchuk, Shynggys Sergazy, Assel Urazbayeva, Alexander Gulyayev, Yerlan Ramankulov, Ivan Kulakov

**Affiliations:** 1 National Laboratory Astana, Nazarbayev University, Kabanbai Batyr Ave. 53, Astan Z05H0P9, Kazakhstan; shynggys.sergazy@gmail.com (S.S.); agulyayev@nu.edu.kz (A.G.); 2 National Center for Biotechnology, 13/5 Kurgalzhynskoe Road, Astana Z05K8D5, Kazakhstan; 3 School of Natural Sciences, Tyumen State University, 15a Perekopskaya St., Tyumen 625003, Russia; i.v.palamarchuk@utmn.ru; 4 School of Science and Technology, Nazarbayev University, Kabanbai Batyr Ave. 53, Astana Z05H0P9, Kazakhstan

**Keywords:** 3-aminopyridin-2(1*H*)-ones, isothiocyanates, thiourea derivatives, *α*-glucosidase inhibition, antidiabetic activity, IC_50_, molecular docking

## Abstract

Reactions with allyl-, acetyl-, and phenylisothiocyanate have been studied on the basis of 3-amino-4,6-dimethylpyridine-2(1*H*)-one, 3-amino-4-phenylpyridine-2-one, and 3-amino-4-(thiophene-2-yl)pyridine-2(1*H*)-one (benzoyl-)isothiocyanates, and the corresponding thioureide derivatives **8-11a-c** were obtained. Twelve thiourea derivatives were obtained and studied for their anti-diabetic activity against the enzyme α-glucosidase in comparison with the standard drug acarbose. The comparison drug acarbose inhibits the activity of α-glucosidase at a concentration of 15 mM by 46.1% (IC_50_ for acarbose is 11.96 mM). According to the results of the conducted studies, it was shown that alkyl and phenyl thiourea derivatives **8,9a-c**, in contrast to their acetyl–(benzoyl) derivatives and **10,11a-c**, show high antidiabetic activity. Thus, 1-(4,6-dimethyl-2-oxo-1,2-dihydropyridin-3-yl)-3-phenylthiourea **9a** has the highest inhibitory activity against the enzyme α-glucosidase, exceeding the activity of the comparison drug acarbose, which inhibits the activity of α-glucosidase by 56.6% at a concentration of 15 mm (IC_50_ = 9,77 mM). 1-(6-methyl-2-oxo 4-(thiophen-2-yl)-1,2-dihydropyridin-3-yl)-3-phenylthiourea **9c** has inhibitory activity against the enzyme α-glucosidase, comparable to the comparison drug acarbose, inhibiting the activity of α-glucosidase at a concentration of 15 mm per 41.2% (IC_50_ = 12,94 mM). Compounds **8a, 8b,** and **9b** showed inhibitory activity against the enzyme α-glucosidase, with a lower activity compared to acarbose, inhibiting the activity of α-glucosidase at a concentration of 15 mM by 23.3%, 26.9%, and 35.2%, respectively. The IC_50_ against α-glucosidase for compounds **8a**, **8b**, and **9b** was found to be 16.64 mM, 19.79 mM, and 21.79 mM, respectively. The other compounds **8c, 10a, 10b, 10c, 11a, 11b**, and **11c** did not show inhibitory activity against α-glucosidase. Thus, the newly synthesized derivatives of thiourea based on 3-aminopyridine-2(1*H*)-ones are promising candidates for the further modification and study of their potential anti-diabetic activity. These positive bioanalytical results will stimulate further in-depth studies, including in vivo models.

## 1. Introduction

It is known by now that the extremely high prevalence of diabetes mellitus is regarded as a global threat. So, if by now, according to the Atlas of Diabetes, approximately 537 million adults worldwide aged 20 to 79 years suffer from diabetes (10.5% of all adults in this age range), then by 2030, approximately 643 million people will suffer from diabetes, and by 2045, their number will increase to 783 million [1,2].

The “diabetes pandemic” requires an intensification of the search for new drug controls.

It is known that thiourea is widely used in the synthesis of heterocyclic compounds, and functional derivatives of the thiourea class exhibit a wide range of pharmacological effects (anticonvulsant, anticancer, antiviral, antifungal, antibacterial), including the hypoglycemic effect [3,4,5,6,7,8]. It is believed that the main pharmacological activity of thiourea derivatives is due to specific interaction with target receptors of proteins or enzymes. Hydrogen bonds of both donor amino groups and the acceptor ionic group may play an important role in this [9].

**Figure 1:**
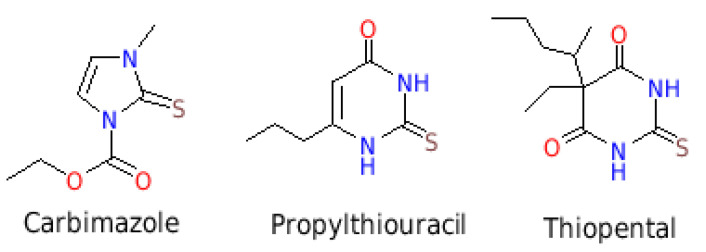
Drugs with structural cyclic thiourea.

Among the most well-known medicinal substances of thiourea, drugs with a cyclic thiourea structure are *Carbimazole* and *Propylthiouracil*, which are used to treat hyperthyroidism (hyperthyroidism), as well as *Thiopental* barbiturate, which is used to induce general anesthesia, treat seizures, and reduce intracranial pressure [10,11,12] (Figure 1).

In particular, the extremely high relevance of the search for new substances with hypoglycemic potential among thiourea derivatives is predetermined by the fact that effective antidiabetic drugs (chlorpropamide, glibenclamide, tolbutamide, glimepiride, glipizide, etc.) have already been created on the basis of sulfonylurea derivatives, the effect of which is based on the stimulation of islet beta cells of the pancreas. At the same time, the main target for sulfonylurea preparations is SUR 1, a receptor for ATP-sensitive potassium ion channels [13,14,15,16,17,18].

**Figure 2:**
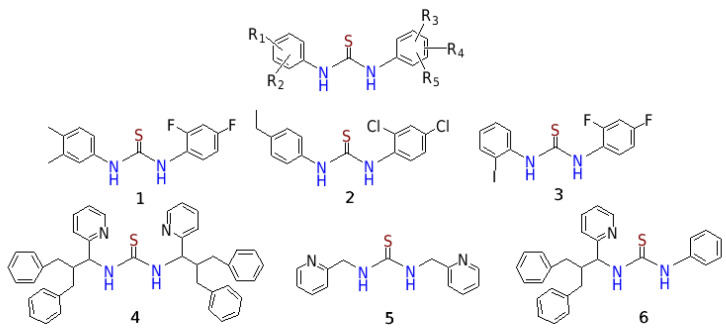
Structural formulas of thiourea derivatives with potential antidiabetic activity.

The search for new antidiabetic agents is also being conducted among conventional thiourea derivatives. Thus, work [19] provides extensive data on the antidiabetic activity of a series of diaryl-substituted thioureas (Figure 2). As a result of bioscreening, the authors identified several diaryl-substituted thioureas 1–3 with the highest inhibitory activity. According to the authors, these deserve attention as potential candidates for the role of hypoglycemic substances.

In [20], S. Naz and co-authors synthesized three thiourea derivatives containing a pyridine aryl backbone (**4**-1,3-bis(2-benzyl-3-phenyl-1-(pyridine-2-yl)propyl)thiourea, **5**-1,3-bis(pyridin-2-ylmethyl) thiourea, and **6**-1-(2-benzyl-3-phenyl-1-(pyridine-2-yl)propyl)-3-phenylthiourea) (Figure 2). They conducted in vivo tests on Swiss albino mice with diabetes caused by a single administration of streptozotocin (the activity of the tested compounds was controlled by the authors through the inhibition of the enzyme glucose-6-phosphatase), which showed high values of enzyme inhibition. Compound **4** had the highest inhibition value and, in addition, proved to be safe for use in animals, without having any toxic or lethal effects, preventing the effects of hyperglycemia and hyperlipidemia, and it also contributed to weight loss in experimental animals.

Previously, we described a method for obtaining 4-aryl(hetaryl)-substituted 3-aminopyridine-2(1*H*)-ones based on the intramolecular cyclization of N-(3-oxoalkenyl)amides [21]. It has been shown that almost all the obtained 3-aminopyridine-2(1*H*)-ones have high antiradical activity. Derivatives of 3-aminopyridine-2(1*H*)-one are of interest as potential biologically active compounds [22,23]. For example, “Amrinone” is an inhibitor of pyridine phosphodiesterase 3, which has cardiotonic and vasodilating effects [24]. Some derivatives of 3-aminopyridine-2(1*H*)-one exhibit antiviral activity, including against the AIDS virus [25,26]. The presence of an “embedded” amino acid fragment makes them attractive building blocks for the synthesis of new derivatives with promising biological applications [27,28].

We also previously showed that the reduction in Schiff bases with sodium borohydride obtained through the condensation of 3-amino-6-methyl-4-phenylpyridine-2(1*H*)-one with aromatic aldehydes leads to the formation of 3-(arylmethyl)-6-methyl-4-phenylpyridine-2(1*H*)-ones, which have also shown high antiradical and cytoprotective activity [29,30], tranquilizing (anxiolytic) activity in the in vivo “dark-light chamber” test, and antidepressant activity in the “Porsolt passive swimming test” [31]. At the same time, several derivatives of 3-(arylmethylamino)-6-methyl-4-phenylpyridine-2(1*H*)-one have been found with higher potential neurotropic activity, higher than in comparison drugs (mexidol and amitriptyline). In addition, conjugates synthesized on the basis of 3-aminopyridine-2(1*H*)-ones containing 1,3,4-thiadiazole cycle have shown their hypoglycemic, antidiabetic potential in inhibiting α-amylase and α-glucosidase and shown excellent antidiabetic activity [32,33] exceeding the effectiveness of the comparison drug acarbose.

Thus, the established high pharmacological potential of new derivatives of 3-aminopyridine-2(1*H*)-ones opens up opportunities and prospects for the search for new substances with a hypoglycemic effect by identifying substances capable of inhibiting the activity of α-amylase and α-glucosidase.

## 2. Results and Discussion

### 2.1. Chemistry

By the time of this study, thioureas obtained on the basis of 3-aminopyridine-2(1*H*)-one were presented only in isolated examples [34,35] and practically not studied biologically.

In order to obtain thioureide and thiourea derivatives of pyridones-2 and their subsequent bioscreening for antidiabetic activity, we synthesized them through the interaction of 3-aminopyridine-2(1*H*)-ones **7a-c** with some isothiocyanates (allylisothiocyanate, phenylisothiocyanate, benzoyl, and acetylisothiocyanate) according to the methods described in [36,37,38].

**Scheme 1:**
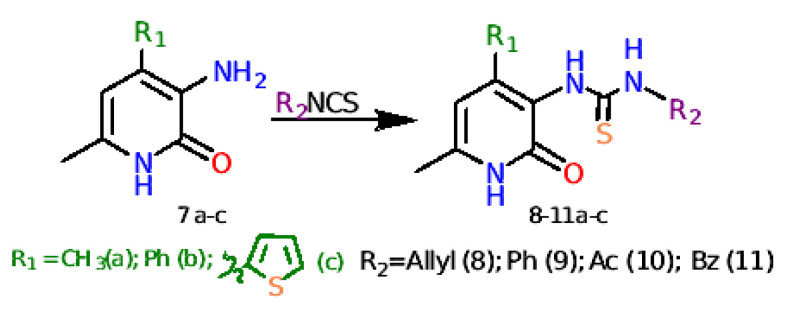
Synthesis of thiourea derivatives **8-11a-c.**

The synthesis of the initial acylisothiocyanates was carried out in situ by heating the corresponding chlorangidrides (benzoyl chloride, acetyl chloride) with potassium thiocyanate in an acetone medium. Further interaction of isothiocyanates with 3-aminopyridine-2-(1*H*)-ones **7a-c** led to the formation of thiourea derivatives **8-11a-c** (Scheme 1).

Thiourea derivatives **8-11a-c** isolated with good yields (70–80%) are fine crystalline powdery substances of white or light beige color, moderately soluble in polar organic solvents (when heated). The structure of the obtained new thiourea derivatives was confirmed via ^1^H and ^13^C NMR spectroscopy and mass spectrometry.

### 2.2. In Vitro α-Glucosidase Inhibition Assay

To check the known literature data on the antidiabetic activity of sulfur-containing derivatives, including thiourea, we carried out screening studies for the presence of antidiabetic activity on compounds **8-11a-c**.

Antidiabetic activity was assessed by the degree of the inhibition of α-glucosidase activity by the test substances.

The study of the *α*-glucosidase activity inhibition degree by the test compounds was performed using a standard method with minor modifications [39].

The results of the study of the inhibitory activity of the test compounds against the *α*-glucosidase enzyme are shown in Table 1.

Table 1 shows the inhibitory activity of tested compounds at a concentration of 15 mM against the *α*-glucosidase enzyme and the concentration at which a 50% inhibition of *α*-glucosidase activity occurs (IC_50_).

**α-Glucosidase Inhibition Assay**

All of the newly synthesized compounds **8-11a-c** were tested for their in vitro α-glucosidase inhibitory activity. The results are summarized in Table 1.

Based on the data presented in Table 1, we see that compound **9a** has the highest inhibitory activity against the α-glucosidase enzyme, exceeding the activity of the acarbose comparison drug, which inhibits the activity of α-glucosidase at a concentration of 15 mM by 56.6%. The calculation of the average inhibitory concentration IC_50_ (mM) of compound **9a** showed a value of 9.77 mM. While the comparison drug acarbose inhibits the activity of α-glucosidase at a concentration of 15 mM by 46.1%, the calculation of the average inhibitory concentration of IC_50_ (mM) acarbose showed a value of 11.96 mM. Compound **9c** has inhibitory activity against the enzyme α-glucosidase, comparable to the comparison drug acarbose, inhibiting the activity of α-glucosidase at a concentration of 15 mM by 41.2%, the calculation of the average inhibitory concentration IC_50_ (mM) of compound 9c showed a value of 12.94 mM. Three more compounds **8a**, **8b**, and **9b** showed inhibitory activity against the enzyme α-glucosidase, inferior in activity to acarbose, inhibiting the activity of α-glucosidase at a concentration of 15 mM by 23.3%, 26.9%, and 35.2%, accordingly. The calculation of the average inhibitory concentration of IC_50_ (mM) with respect to α-glucosidase for compounds **8a**, **8b**, and **9b** showed values of 16.64 mM, 19.79 mM, and 21.79 mM, respectively. The remaining compounds **8c**, **10a**, **10b**, **10c**, **11a**, **11b**, and **11c** showed no inhibitory activity against α-glucosidase.

### 2.3. Molecular Docking

In order to obtain an idea of the protein–ligand interactions of synthesized thiourea derivatives **8-11a-c** in the active center of the enzyme, studies were conducted using molecular docking. 

Despite the fact that out of the 12 synthesized thiourea derivatives, only five (**8a**, **8b**, **9a-c**) demonstrated high inhibitory activity against the enzyme α-glucosidase, all compounds were selected for further calculations.

Further, the structures of the molecules were docked with the active center of proteins (PDB ID: 3A4A) [40] and (PDB ID: 5NN8) [41], since they play an important role in maintaining glucose levels in the body.

Molecular modeling was performed using the AutoDock Vina software package [42] (https://vina.scripps.edu/ accessed on 20 May 2024). Three-dimensional (3D) structures were obtained from the RCSB Protein Data Bank [43]. The chemical structures of the studied compounds were drawn using the ChemOffice software (Chem Draw 16.0), and energy minimization for three-dimensional stabilization of the structure was performed using ChemBio3D Ultra 14.0. Protein structures were prepared for docking by removing a water molecule and a native ligand; hydrogen atoms were added to the standard geometry before docking, and the structures were converted to pdbqt format using the AutoDock MGL software package [44] (https://ccsb.scripps.edu/mgltools/ accessed on 20 May 2024). A program with the AutoDock Vina graphical user interface was used to set up a grid for modeling docking. The grid was installed in such a way that it surrounded the area of interest of the macromolecule. Active sites of the corresponding proteins were predicted using the CASTp server [45]. For the isomaltase enzyme (PDB ID: 3A4A) [40], the coordinates of the active site grid were X = 18.70, Y = −6.80, and Z = 23.50 (size: 22 × 18 × 22 Å) [46]; for the α-glucosidase enzyme (PDB ID: 5NN8) [41], the coordinates of the active site grid were X = −11.00, Y = −38.95, and Z = 94.39 (size: 25Χ25Χ25 Ε) [47]. During the docking process, no more than nine conformations were considered for each ligand. The conformations with the most favorable (lowest) free binding energy were selected for the analysis of interactions between the target receptor and ligands using the Discovery Studio 2015 Visualizer software package [48].

The control docking procedure was conducted using co-crystallized ligands to verify the docking parameters, followed by their extraction and redocking into the same binding pockets. The molecular docking procedure was validated as effective and reliable, with a root mean square deviation (RMSD) of less than 1.5 Å. The redocked poses nearly overlap with the co-crystallized conformations. Docking parameters are considered acceptable if the RMSD of the docking ligand relative to the crystallized one is less than 1.5 Å. [49].

The results of the molecular docking show that the affinity of the interaction of the studied compounds, **8a-c**, **9b,c**, **10a-c**, and **11b**, with the selected protein receptors (PDB ID: 3A4A, PDB ID: 5NN8) did not exceed the affinity of the interaction of these proteins with acarbose used as a comparison (Table 2).

On the other hand, compounds **9a** and **11b** showed high binding ability to selected protein receptors, which is partly consistent with the results of in vitro tests assessing the inhibitory activity of α-glucosidase.

The lack of actual biological activity in the acyl derivatives of thioureas **10a-c** and **11a-c**, despite the molecular docking showing very good docking results, can be explained by the poor solubility of these compounds under the conditions of the biological experiment with the enzyme α-glucosidase.

Since, according to the results of docking, compounds **9a** and **9c** showed better results in binding energy, we describe in more detail their interaction with two receptor proteins.

**Figure 3:**
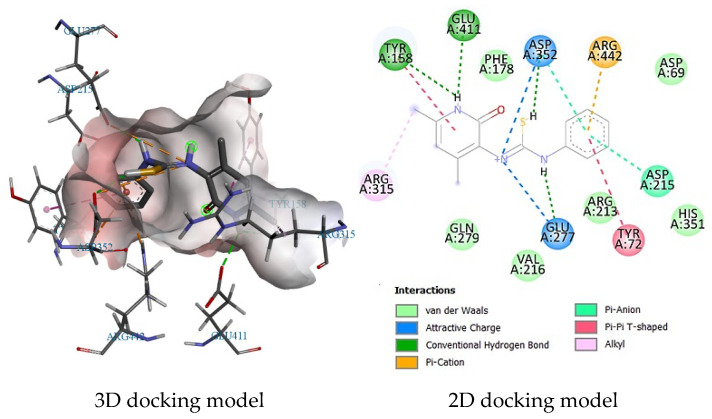
Complex of **9a** with isomaltase (PDB ID: 3A4A).

Thus, an analysis of the interaction of compound **9a** with a protein receptor (PDB identifier: 3A4A) showed that the resulting complex has a high binding energy in the active site of the protein (−8.6 kcal/mol) of formation due to four strong hydrogen bonds of NH and SH groups with amino acid residues: TYR158, GLU411, GLU277, and ASP352. In addition, the amino acids TYR158 and TYR72 form a π-π T-shaped interaction with the π systems of pyridone phenyl and rings, respectively. Also, the amino acid residues ASP352 and ASP215 interact with the π-system of the phenyl ring through the π-anionic bond. In addition, the presence of six Van der Waals interactions was recorded: GLN279, VAL216, ARG 213, HIS351, PHE178, and ASP69 (Figure 3).

**Figure 4:**
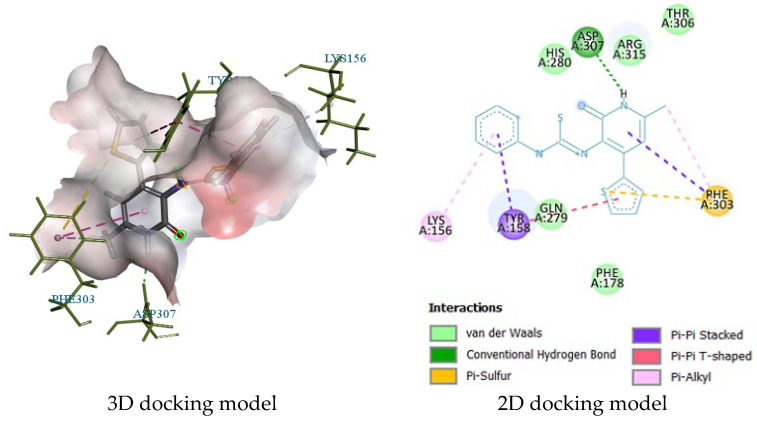
Complex of **9c** with isomaltase (PDB ID: 3A4A).

Compound **9c** has a binding energy (−8.2 kcal/mol) in the active site of the protein due to the formation of two π-π interactions; the π-systems of the phenyl and pyridone rings with amino acid residues TYR158 and PHE303, respectively, are composed. It has also been shown that the New Hampshire group interacts with the amino acid residue of ASP307 stable through hydrogen bonding. A π-π T-shaped space appeared between the π-system T-shaped column and the release of amino acids TYR158. In addition, five Van der Waals interactions are formed; HIS280, ARG315, THR306, GLN279, and PHE178 can be stained (Figure 4).

**Figure 5:**
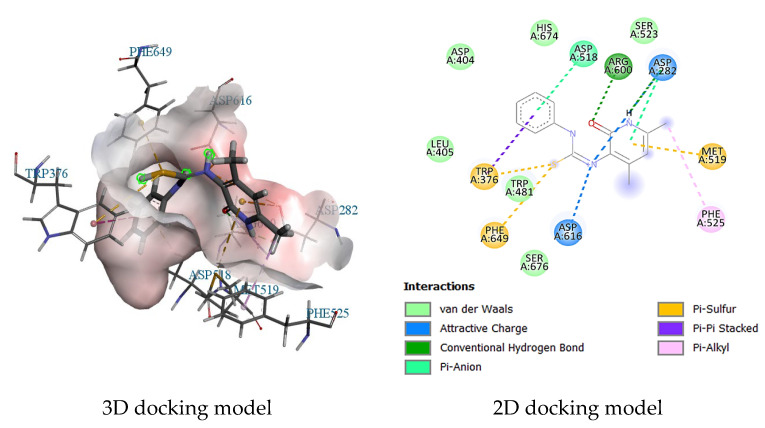
Complex of **9a** with α-glucosidase (PDB ID: 5NN8).

Compound **9a** demonstrated high binding affinity to the 5NN8 protein (−7.5 kcal/mol) due to the formation of two hydrogen bonds between the NH group and the oxygen atom of the pyridone ring with amino acid residues ASP282 and ARG600, respectively. There is also a π-π stacked interaction between the amino acid residue TRP376 and the π-system of the phenyl ring. In addition, the S atom and the pyridone ring form three π-sulfur interactions with the residues PHE649, TRP376, and MET519. Other amino acid residues such as LEU405, ASP404, HIS674, SER523, TRP481, and SER676 form Van der Waals interactions (Figure 5).

**Figure 6:**
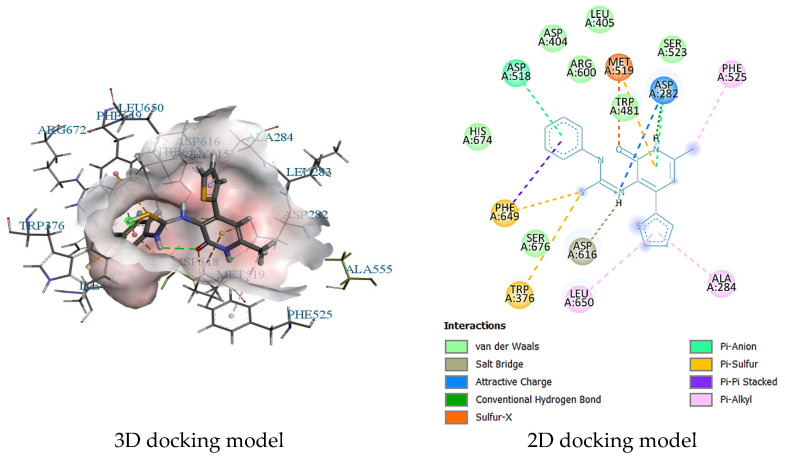
Complex of **9c** with α-glucosidase (PDB ID: 5NN8).

The docking analysis showed that compound **9b** has a binding affinity to the selected protein receptor (−7.1 kcal/mol). As shown in Figure 6, compound **9c** forms one strong hydrogen bond between the NH group of the pyridine ring with the amino acid residue ASP282. The formation of two π-anion interactions is realized by binding the π-system of the phenyl and pyridone rings to the amino acid residues ASP518 and ASP282, respectively. In addition, as in the case of compound **9a**, three π-sulfur interactions are formed between the S atom and the pyridone ring with residues PHE649, TRP376, and MET519. The presence of a family of Van der Waals interactions with amino acid residues LEU405, ASP404, HIS674, SER523, TRP481, ARG600, and SER676 was also recorded.

Thus, according to the results of the molecular docking of phenylthiourea **9a**, **c** showed a good correlation with the conducted in vitro tests.

The docking results and interactions with target proteins for the other compounds are provided in the Supplementary Materials (Tables S1 and S2).

## 3. Experimental Procedures

### 3.1. Materials and Methods

The description of this section (figures of spectrums) is included as Supplementary Materials.

^1^H and ^13^C NMR spectra were recorded on a Bruker DRX400 (400 and 100 MHz, respectively) and Bruker AVANCE 500 (500 and 125 MHz, respectively), and Magritek spinsolve 80 carbon ultra (81 and 20 MHz, respectively) instruments were used using the DMSO-*d*_6_ internal standard with TMS or residual solvent signals (2.49 and 39.9 ppm ^1^H and for ^13^C nuclei in DMSO-*d*_6_).

Samples were analyzed by HPLC-MS on an Agilent 1260 Infinity II chromatograph coupled to an Agilent 6545 LC/Q-TOF high-resolution mass spectrometer with a Dual AJS ESI ionization source operating in positive ion mode using the following parameters: capillary voltage: 4000 V; spray pressure: 20 (psi); drying gas: 10 *l*/min; gas temperature: 325 °C; sheathed gas flow: 12 *l*/min; shielding gas temperature: 400 °C; nozzle voltage: 0 V; fragmentation voltage: 180 V; skimmer voltage: 45 V; and octopole RF: 750 V. Mass spectra with LC/MS accuracy were recorded in the range 100–1000 *m*/*z*, scan rate: 1.5 spectrum/s.

Chromatographic separation was carried out on columns: ZORBAX RRHD Eclipse Plus C18 (2.1 × 50 mm, particle size 1.8 µm). The column temperature during the analysis was maintained at 35 °C. The mobile phase was formed by eluents A and B. In the positive ionization mode, 0.1% formic acid solution in deionized water was used as eluent A, and 0.1% formic acid solution in acetonitrile was used as eluent B. Chromatographic separation was performed with elution according to the following scheme: 0–10 min 95% A, 10–13 min 100% B, and 13–15 min 95% A. The flow of the mobile phase was maintained at 400 μL/min throughout the analysis. In all experiments, the sample injection volume was 1 μL. The sample was prepared by dissolving the entire sample (in 1000 μL) in methanol (for HPLC). Sample dilution was carried out immediately before analysis.

The recorded data were processed using Agilent MassHunter 10.0 software.

Melting points were determined using a Stuart SMP10 hot bench. The monitoring of the reaction course and the purity of the products were carried out by TLC on Sorbfil plates and visualized using iodine vapor or UV light.

### 3.2. Synthesis of Thiourea Derivatives: The General Methodology

Method A. To the solution of the corresponding 3-amino-4,6-dimethylpyridin-2(1*H*)-one **7a-c** (1.0 mmol) in a mixture of DMF–methylene chloride (2:1), 1.2 mmol phenyl isothiocyanate (for compounds **9a-c**) was added dropwise. The reaction mixture was stirred for 10–15 h at room temperature. The resulting precipitate was cooled, filtered, washed with cold acetone, dried, and recrystallized from a mixture of solvents 2-propanol–hexane (2:1) or DMF–2-propanol (for **9c**).

Method B. A mixture of ammonium thiocyanate (1.2 mmol), acetyl chloride (for compounds **10a-c**), or benzoyl chloride (for compounds **11a-c**) in 20 mL of acetone was heated with reflux and stirring for 2 h. The resulting precipitate of KCl was filtered off and immediately added to a solution of 1 mmol of the corresponding 3-aminopyridin-2(1*H*)-one (**7a-c**) in 10 mL of acetone and stirred for an additional 3 h. The reaction mixture with the precipitated solid was cooled, filtered, washed with acetone, and dried. After recrystallization from a mixture of 2-propanol–DMF (2:1), compounds **10-11a-c** were obtained.

The physicochemical constants and spectral characteristics of thioureas 1-allyl-3-(4,6-dimethyl-2-oxo-1,2-dihydropyridin-3-yl)thiourea (**8a**), 1-allyl-3-(6-methyl-2-oxo-4-phenyl-1,2-dihydropyridin-3-yl)thiourea (**8b**), N-((4,6-dimethyl-2-oxo-1,2-dihydropyridin-3-yl)carbamothioyl)benzamide (**11a**), and N-((6-methyl-2-oxo-4-phenyl-1,2-dihydropyridin-3-yl)carbamothioyl)benzamide (**11b**) were described by us in [33].

1-Allyl-3-(6-methyl-2-oxo-4-(thiophen-2-yl)-1,2-dihydropyridin-3-yl)thiourea **(8c).** Yield 360 mg (59%), M.p.: = 289–292 °C. ^1^H NMR (400 MHz, DMSO-d6) δ ppm (*J*, Hz): 2.20 (s, 3H, 6-CH_3_); 4.05 (br. s, 2H, CH_2_); 4.98 (br. s., 1H, C=H_a_); 5.11 (br. d, *J* = 15.1 Hz, 1H, C=H_b_); 5.76 (br. s., 1H, -CH=CH_a_H_b_); 6.45 (s, 1H, H-5); 7.14 (d, *J* = 4.1 Hz, 1H, H-4 thiophene); 7.61 (br. s. 1H, NH-CH_2_); 7.66 (d, *J* = 3.7 Hz, 1H, H-3 thiophene); 7.71 (d, *J* = 4.6 Hz, 1H, H-5 tiophene); 8.52 (br. s., 1H, NHCS); 11.64 (br. s., 1H, NHCO). ^13^C NMR (101 MHz, DMSO-d6) δ ppm 18.5 (CH_3_); 46.3 (NH-CH_2_); 102.6; 114.9 (=CH_2_); 120.5; 126.9 (C-3 thiophene); 128.7 (C-4 thiophene); 130.3 (C-5 thiophene); 135.1 (CH_2_C=); 137.3; 141.0; 143.3; 161.0; 189.0 (CS).

1-(4,6-Dimethyl-2-oxo-1,2-dihydropyridin-3-yl)-3-phenylthiourea **(9a).** Yield: 0.180 g (66%), white powder, M.p.: 140–143 °C. ^1^H NMR (500 MHz, DMSO-d6) δ ppm (*J*, Hz): 2.05 (s, 1H, CH_3_); 2.13 (s, 1H, CH_3_); 5.90 (s, 1H, H-5); 7.09 (t, 1H, *J =* 7.2 Hz, H-4 Ph); 7.30 (t, 2H, *J =* 7.6 Hz, H-3,5 Ph); 7.50 (d, 2H, *J =* 7.3 Hz, H-3,5 Ph); 8.68 (br. s., 1H, NHPh); 9.57 (br. s., 1H, NHCS); 11.63 (br. s., 1H, NHCO). ^13^C NMR (125 MHz, DMSO-d6) δ ppm 18.2 (CH_3_); 18.2 (CH_3_); 106.6 (C-5); 124.1 (2C Ph); 125.9 (C Ph); 128.3 (2C Ph); 129.9; 139.7; 142.3; 147.9; 160.4; 180.7. HRMS *m*/*z*: calcd for C_14_H_16_N_3_OS^+^ [M + H]^+^: 274.1009; found: 274.0099.

1-(6-Methyl-2-oxo-4-phenyl-1,2-dihydropyridin-3-yl)-3-phenylthiourea **(9b).** Yield: 0.232 g (69%), white powder, M.p.: 141–144 °C. ^1^H NMR (81 MHz, DMSO-d6) δ ppm (*J*, Hz): 2.22 (s, 1H, CH_3_); 6.03 (s, 1H, H-5); 7.14–7.43 (m, 10H, H-2,3,4,5,6 Ph, H-2,3,4,5,6 Ar); 8.67 (br. s., 1H, NHPh); 9.51 (br. s, 1H, NHCS); 11.83 (br. s., 1H, NHCO). ^13^C NMR (20 MHz, DMSO-d6) δ ppm 18.4 (CH_3_); 105.8 (C-5); 123.6 (2C Ph); 128.2 (8C Ph); 137.6; 139.6; 143.3; 149.0; 160.8; 162.3; 181.2. HRMS *m*/*z*: calcd for C_19_H_18_N_3_OS^+^ [M + H]^+^: 336.1165; found: 336.1175.

1-(6-Methyl-2-oxo-4-(thiophen-2-yl)-1,2-dihydropyridin-3-yl)-3-phenylthiourea **(9c).** Yield: 0.260 g (76%), white powder, M.p.: 145–147 °C. ^1^H NMR (81 MHz, DMSO-d6) δ ppm (*J*, Hz): 2.22 (s, 1H, CH_3_); 6.47 (s, 1H, H-5); 7.09–7.76 (m, 8H, H-2,3,4,5,6 Ph, H-3,4,5 thiophene); 8.76 (s, 1H, NHPh); 9.73 (br. s, 1H, NHCS); 11.69 (br. s., 1H, NHCO). ^13^C NMR (20 MHz, DMSO-d6) δ ppm 18.5 (CH_3_); 102.8 (C-5); 124.3 (2C Ph); 126.9 (1C thiophene); 128.2 (5C Ph); 128.7 (1C thiophene); 130.2 (1C thiophene); 137.4; 139.6; 141.4; 143.0; 160.9; 162.3; 181.6. HRMS *m*/*z*: calcd for C_17_H_16_N_3_OS_2_^+^ [M + H]^+^: 342.0729; found: 342.0735.

N-((4,6-Dimethyl-2-oxo-1,2-dihydropyridin-3-yl)carbamothioyl)acetamide **(10a).** Yield: 0.127 g (53%), light beige powder, M.p.: 273–275 °C. ^1^H NMR (500 MHz, DMSO-d6) δ ppm (*J*, Hz): 1.99 (s, 3H, CH_3_); 2.12 (s, 6H, 2CH_3_); 5.90 (s, 1H, H-5); 11.42 (br. s., 1H, NHCS); 11.44 (br. s., 1H, NHCO); 11.69 (br. s., 1H, NHCO). ^13^C NMR (125 MHz, DMSO-d6) δ ppm 18.18 (CH_3_); 18.21 (CH_3_); 23.7 (CH_3_); 106.6 (C-5); 123.1; 143.0; 147.6; 159.5; 172.4; 180.7. HRMS *m*/*z*: calcd for C_10_H_14_N_3_O_2_S^+^ [M + H]^+^: 240.0801; found: 240.0810.

N-((6-Methyl-2-oxo-4-phenyl-1,2-dihydropyridin-3-yl)carbamothioyl)acetamide **(10b).** Yield: 0.168 g (56%), light beige powder, M.p.: 249–251 °C. ^1^H NMR (500 MHz, DMSO-d6) δ ppm (*J*, Hz): 2.05 (s, 3H, CH_3_); 2.21 (s, 3H, CH_3_); 6.02 (s, 1H, H-5); 7.35–7.39 (m, 3H, H-3,4,5 Ph); 7.42–7.45 (m, 2H, H-2,6 Ph); 11.29 (br. s., 1H, NHCS); 11.34 (br. s., 1H, NHCO); 11.91 (br. s, 1H, NHCO). ^13^C NMR (125 MHz, DMSO-d6) δ ppm 18.4 (CH_3_); 23.6 (CH_3_); 105.5 (C-5); 122.1; 127.6 (2C Ph); 128.2 (2C Ph); 128.5; 137.1; 144.0; 148.9; 159.5; 172.2; 181.4. HRMS *m*/*z*: calcd for C_15_H_16_N_3_O_2_S^+^ [M + H]^+^: 302.0958; found: 302.0963.

N-((6-Methyl-2-oxo-4-(thiophen-2-yl)-1,2-dihydropyridin-3-yl)carbamothioyl)acetamide **(10c).** Yield: 0.169 g (55%), light beige powder, M.p.: 252–254 °C. ^1^H NMR (500 MHz, DMSO-d6) δ ppm (*J*, Hz): 2.16 (s 3H, CH_3_’); 2.20 (s, 3H, CH_3_); 6.46 (s, 1H, H-5); 7.14 (dd, 1H, *J =* 5.0 Hz, *J =* 3.7 Hz, H-4 thiophene); 7.64 (dd, 1H, *J =* 3.8 Hz, *J =* 1.1 Hz, H-3 thiophene); 7.72 (dd, 1H, *J =* 5.1 Hz, *J =* 1.0 Hz, H-5 tiophene); 11.47 (br. s, 1H, NHCS); 11.56 (br. s, 1H, NHCO); 11.77 (br. s, 1H, NHCO). ^13^C NMR (125 MHz, DMSO-d6) δ ppm 18.5 (CH_3_); 23.7 (CH_3_); 102.6 (C-5); 120.2; 127.2 (C-3 thiophene); 129.00 (C-4 thiophene); 130.3 (C-5 thiophene); 136.8; 140.6; 143.6; 159.7; 172.5; 182.2. HRMS *m*/*z*: calcd for C_13_H_14_N_3_O_2_S_2_^+^ [M+H]^+^: 308.0522; found: 308.0530.

N-((4,6-Dimethyl-2-oxo-1,2-dihydropyridin-3-yl)carbamothioyl)benzamide **(11a).** Yield: 0.682 g (76%), light-yellow, finely crystalline powder, M.p.: 230–232 °C. ^1^H NMR (400 MHz, DMSO-d6) δ ppm (*J*, Hz): 2.07 (s, 3H, 4-CH_3_); 2.16 (s, 3H, 6-CH_3_); 5.92 (s, 1H, H-5); 7.53 (t, *J* = 7.6 Hz, 2H, H-3,5 Ph); 7.65 (t, *J* = 7.6 Hz, 1H, H-4 Ph); 7.98 (d, *J* = 7.8 Hz, 2H, H-2,6 Ph); 11.42 (br. s, 1H, NHCS); 11.62 (br. s, 1H, NHCO); 11.64 (br. s, 1H, NHCO). ^13^C NMR (100 MHz, DMSO-d6) δ ppm 18.2 (4,6-CH_3_); 106.6 (C-5); 123.4 (C-4); 128.4 (C-2,6 Ph); 128.6 (C-3,5 Ph); 132.1 (C-1 Ph); 133.1 (C-4 Ph); 143.1 (C-3); 147.6 (C-6); 159.5 (C-2); 168.3 (CO); 180.9 (CS). HRMS *m*/*z*: calcd for C_15_H_16_N_3_O_2_S^+^ [M + H]^+^: 302.0958; found: 302.0963.

N-[(6-Methyl-2-oxo-4-phenyl-1,2-dihydropyridin-3-yl)carbamothioyl]benzamide **(11b).** Yield: 0.244 g (67%), light-yellow, finely crystalline powder, M.p.: 218–220 °C. ^1^H NMR (400 MHz, DMSO-d6) δ ppm (*J*, Hz): 2.24 (s, 3H, 6-CH_3_); 6.05 (s, 1H, H-5); 7.34–7.41 (m, 3H, H-2,4,6 Ph); 7.47–7.51 (m, 4H, H-3,5 Ph, H-3,5 Ph’); 7.62 (t, *J* = 7.4 Hz, 1H, H-4 Ph’); 7.91 (d, *J* = 7.2 Hz, 2H, H-2,6 Ph’); 11.41 (br. s, 1H, NHCS); 11.63 (br. s, 1H, NHCO); 11.92 (br. s, 1H, NHCO). ^13^C NMR (100 MHz, DMSO-d6) δ ppm 18.9 (6-CH_3_); 106.0 (C-5); 122.8 (C-1 Ph); 128.1 (C-2,6 Ph); 128.7 (C-3,5 Ph); 128.9 (C-4 Ph, C-2,6 Bz); 129.0 (C-3,5 Bz); 132.4 (C-1 Bz); 133.6 (C-4 Bz); 137.6 (C-4); 144.5 (C-3); 149.4 (C-6); 160.0 (C-2); 168.4 (CO); 182.0 (CS). HRMS *m*/*z*: calcd for C_20_H_18_N_3_O_2_S^+^ [M + H]^+^: 364.1114; found: 364.1113.

N-((6-Methyl-2-oxo-4-(thiophen-2-yl)-1,2-dihydropyridin-3-yl)carbamothioyl)benzamide **(11c).** Yield: 0.314 g (85%), light beige powder, M.p.: 245–249 °C. ^1^H NMR (500 MHz, DMSO-d6) δ ppm (*J*, Hz): 2.23 (s, 3H, CH_3_); 6.50 (s, 1H, H-5); 7.15 (dd, 1H, *J =* 4.9 Hz, *J =* 4.1 Hz, H-4 thiophene); 7.55 (t, 2H, H-3,5 Ph); 7.66 (m, 1H, H-4 Ph); 7.69 (d, 1H, H-3 thiophene); 7.72 (d, 1H, H-5 thiophene); 8.01 (d, 2H, H-2,6 Ph); 11.76 (br. s, 2H, NHCS, NHCO); 11.81 (br. s, 1H, NHCO). ^13^C NMR (125 MHz, DMSO-d6) δ ppm 18.5 (CH_3_); 102.7 (C-5); 120.4; 127.2; 128.5 (2C Ph); 128.7 (2C Ph); 129.0; 130.2; 132.0; 133.2; 136.8; 140.6; 143.6; 159.7; 168.2; 182.4. HRMS *m*/*z*: calcd for C_18_H_15_N_3_O_2_S_2_^+^ [M + H]^+^: 370.0678; found: 370.0670.

### 3.3. Biological Tests

#### In Vitro Assay of α-Glucosidase Inhibitory Activity

Antidiabetic activity was assessed by the degree of inhibition of *α*-glucosidase activity by the test substances. The α-glucosidase enzyme determines the extent to which glucose enters the bloodstream from the gastrointestinal tract. The inhibition of this enzyme can be useful for lowering postprandial glucose levels [39]. α-Glucosidase inhibitory activity was assayed using 0.1 M phosphate buffer (pH 6.8) at 37 °C. The enzyme (α-glucosidase from *Saccharomyces cerevisiae*, Sigma-Aldrich, St. Louis, MO, USA, 1.0 U/mL) in phosphate-buffered saline was incubated with various concentrations of test compounds at 37 °C for 15 min. All the studied substances were dissolved in 40% DMSO. Then, 5 mM p-nitrophenyl α-d-glucopyranoside was added to the mixture as a substrate. The mixture was incubated at 3 °C for 20 min. The absorbance was measured spectrophotometrically at 405 nm. The sample solution was replaced by 40% DMSO as a control. Acarbose was used as a positive control. All samples were studied in triplets.

Inhibitory activity was expressed as a percentage (%) according to the degree of inhibition of α-glucosidase in comparison with the negative control. It was calculated using the following formula:

Inhibitory activity (%) = (1 − As/Ac) × 100%, where As is the optical density of the test compound, and Ac is the optical density of control.

The IC_50_ was determined from the graph of enzyme activity changes depending on the sample concentration.

The statistical processing of the results was carried out using the “Excel 2019” program. The obtained results are presented as “mean ± standard error of the mean”.

## 4. Conclusions

Thus, based on 3-aminopyridine-2-(1*H*)-ones **7a-c**, we obtained thioureide derivatives **8-11a-c** and studied their inhibitory antidiabetic activity. According to the results of the conducted studies, it was shown that alkyl and phenyl thiourea derivatives **8,9a-c** show high and moderate antidiabetic activity, in contrast to their acetyl and benzoyl derivatives **10, 11a-c.**

Thus, compound **9a** has the highest inhibitory activity against the enzyme α-glucosidase, exceeding the activity of the comparison drug acarbose, inhibiting the activity of α-glucosidase at a concentration of 15 mM by 56.6%; IC_50_ for **9a** is 9.77 mM. While the comparison drug acarbose inhibits the activity of α-glucosidase at a concentration of 15 mM by 46.1%, the IC_50_ for acarbose is 11.96 mM. Compound **9c** has inhibitory activity against the enzyme α-glucosidase, comparable to the comparison drug acarbose, inhibiting the activity of α-glucosidase at a concentration of 15 mM by 41.2%, the IC_50_ of compound **9c** is 12.94 mM. Compounds **8a, 8b**, and **9b** showed inhibitory activity against the enzyme α-glucosidase, inferior in activity to acarbose, inhibiting the activity of α-glucosidase at a concentration of 15 mM by 23.3%, 26.9%, and 35.2%, respectively. The IC50 for α-glucosidase for compounds **8a**, **8b**, and **9b** is 16.64 mM, 19.79 mM, and 21.79 mM, respectively. The remaining compounds **8c**, **10a**, **10b**, **10c**, **11a**, **11b**, and **11c** showed no inhibitory activity against α-glucosidase.

The results of molecular docking show that the affinity of the interaction of the studied compounds **8a-c**, **9b,c**, **10a-c,** and **11b** with the selected protein receptors (PDB ID: 3A4A, PDB ID: 5NN8) did not exceed the affinity of the interaction of these proteins with acarbose used as a comparison.

The results of molecular docking show that the affinity of the interaction of some of the studied compounds **8a-c**, **9b,c**, **10a-c**, and **11b** with selected protein receptors (PDB ID: 3A4A, PDB ID: 5NN8) was either lower or at the level of the affinity of the interaction of these proteins with acarbose, used as a comparison and, was slightly higher than it for compounds **9a**, **11a**, and **11c**. 

The lack of actual biological activity in the acyl derivatives of thioureas **10a-c** and **11a-c**, despite the molecular docking showing very good docking results, can be explained by the poor solubility of these compounds under the conditions of the biological experiment with the enzyme α-glucosidase.

Taking into account all the data obtained, the new thiourea derivatives synthesized by us based on 3-aminopyridine-2(1*H*)-ones are very promising objects for the further study of their possible antidiabetic activity.

## Figures and Tables

**Table 1:** Inhibitory activity of the test compounds **8-11a-c** against the α-glucosidase enzyme.

Compound	Structure	Inhibitory Activity of Test Compounds at a Concentration of 15 mM Against the α-Glucosidase Enzyme (%)	IC_50_ (mM)
**8a**	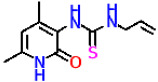	23.3 ± 1.7	16.64 ± 1.57
**8b**	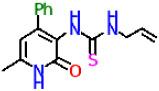	26.9 ± 2.5	19.79 ± 2.55
**8c**	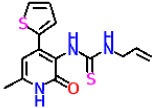	No activity	No activity
**9a**	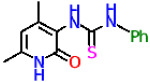	56.6 ± 2.2	9.77 ± 0.83
**9b**	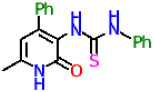	35.2 ± 3.4	21.79 ± 2.65
**9c**	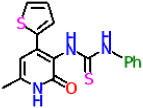	41.2 ± 2.5	12.94 ± 1.08
**10a**	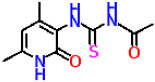	No activity	No activity
**10b**	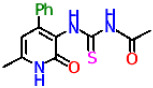	No activity	No activity
**10c**	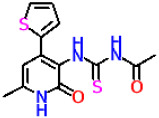	No activity	No activity
**11a**	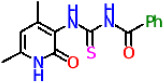	No activity	No activity
**11b**	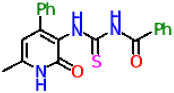	No activity	No activity
**11c**	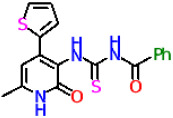	No activity	No activity
Acarbose		46.1 ± 4.6	11.96 ± 1.34

**Table 2:** Binding affinity (kcal/mol) of thiourea derivatives **8a-c-11a-c** and acarbose in the active centers of the binding pocket of the isomaltase (PDB: 3A4A) and α-glucosidases (PDB: 5NN8) enzymes.

	Receptor	3A4A	5NN8
Ligand			
Acarbose		−8.4	−8.0
**8a**		−6.8	−5.8
**8b**		−7.8	−6.5
**8c**		−6.6	−6.0
**9a**		−8.6	−8.2
**9b**		−7.6	−7.2
**9c**		−8.2	−7.8
**10a**		−6.9	−6.2
**10b**		−7.6	−7.8
**10c**		−6.9	−6.8
**11a**		−8.5	−7.6
**11b**		−8.3	−7.8
**11c**		−8.6	−7.2

## Data Availability

Data are contained within the article and Supplementary Materials.

## Supplementary Materials

The following supporting information can be downloaded at https://www.mdpi.com/article/10.3390/molecules29153627/s1; Experimental Procedures, Spectroscopic and physical data; ^1^H and ^13^C NMR spectra; Mass spectra; Tables S1–S2: Molecular docking data.
