# Supplementary material for: Synthesis, Computational Study, and In Vitro α-Glucosidase Inhibitory Action of Thiourea Derivatives Based on 3-Aminopyridin-2(1H)-Ones
Source: Molecules. 2024 Jul 31;29(15):3627. doi: 10.3390/molecules29153627 (PMC11314129; doi:10.3390/molecules29153627)
Supplement: Supplementary file 1 [file molecules-29-03627-s001.zip › molecules-3096751-supplementary.pdf]

Supporting Information

Synthesis, Computational Study, and In Vitro α-Glucosidase Inhibitory Action of Thiourea Derivatives Based on 3-Aminopyridin-2(1H)-Ones

Zarina Shulgau 1,2,*, Irina Palamarchuk 3, Shynggys Sergazy 1,2, Assel Urazbayeva 1,2, Alexander Gulyayev 1,2, Yerlan Ramankulov 4 and Ivan Kulakov 1,3,*

1 National Laboratory Astana, Nazarbayev University, Kabanbai Batyr Ave. 53, Astan Z05H0P9, Kazakhstan
2 National Center for Biotechnology, 13/5 Kurgalzhynskoe Road, Astana Z05K8D5, Kazakhstan
3 School of Natural Sciences, Tyumen State University, 15a Perekopskaya St., Tyumen 625003, Russia
4 School of Science and Technology, Nazarbayev University, Kabanbai Batyr Ave. 53,
Astana Z05H0P9, Kazakhstan

* Correspondence: zarina.shulgau@icloud.com (Z.S.); i.v.kulakov@utmn.ru (I.K.); Tel.: +7-777-708-0837 (Z.S.)

Table of Contents

Experimental Procedures …….......... 1
1. Materials and Methods.......... 1
2. Synthesis of thiourea derivatives……….......... 2
3. Author Contributions.......... 6
4. Copies of NMR Spectra of Products.......... 7
5. Copies of MS Spectra of Products.......... 19
6. Table 1. Complexes between synthesized derivatives 8-11(a-c) and active sites of proteins (PDB: 3A4A, 5NN8) 22
7. Table 2. Basic amino acid interactions and H-bonds 29

Experimental Procedures

1. Materials and Methods

^{1}H and ^{13}C NMR spectra were recorded on a Bruker DRX400 (400 and 100 MHz, respectively), Bruker AVANCE 500 (500 and 125 MHz, respectively) and Magritek spinsolve 80 carbon ultra (81 and 20 MHz, respectively) instruments using DMSO-d6 the internal standard was TMS or residual solvent signals (2.49 and 39.9 ppm ^{1}H and for ^{13}C nuclei in DMSO-d6).
Sample were analyzed by HPLC-MS on an Agilent 1260 Infinity II chromatograph coupled to an Agilent 6545 LC/Q-TOF high-resolution mass spectrometer with a Dual AJS ESI ionization source operating in positive ion mode using the following parameters: capillary voltage: 4000 V; spray pressure: 20 (psi); drying gas: 10 *l*/min; gas temperature: 325ºC; sheathed gas flow: 12 *l*/min; shielding gas temperature: 400°C; nozzle voltage: 0 V, fragmentation voltage: 180 V; skimmer voltage: 45 V; octopole RF: 750 V. Mass spectra with LC/MS accuracy were recorded in the range 100-1000 m/z, scan rate 1.5 spectrum/s.
Chromatographic separation was carried out on columns: ZORBAX RRHD Eclipse Plus C18 (2.1 x 50 mm, particle size 1.8 μm). The column temperature during the analysis was maintained at 35°C. The mobile phase was formed by eluents A and B. In the positive ionization mode, 0.1% formic acid solution in deionized water was used as eluent A, and 0.1% formic acid solution in acetonitrile was used as eluent B. Chromatographic separation was performed with elution according to the following scheme: 0-10 min 95% A, 10-13 min 100% B, 13-15 min 95% A. The flow of the mobile phase was maintained at 400 μL/min throughout the analysis. In all experiments, the sample injection volume was 1 μL. The sample was prepared by dissolving the entire sample (in 1000 μL) in methanol (for HPLC). Sample dilution was carried out immediately before analysis.
The recorded data were processed using Agilent MassHunter 10.0 software.
Melting points were determined using a Stuart SMP10 hot bench. Monitoring of the reaction course and the purity of the products was carried out by TLC on Sorbfil plates and visualized using iodine vapor or UV light.

The general procedure for obtaining thiourea derivatives is as follows:

Metod A. To the solution of the corresponding 3-amino-4,6-dimethylpyridin-2(1*H*)-one **7a-c** (1.0 mmol) in a mixture of DMF: methylene chloride (2:1), 1.2 mmol phenyl isothiocyanate (for compounds **9a-c**) is added dropwise. The reaction mixture is stirred for 10-15 hours at room temperature. The resulting precipitate is cooled, filtered, washed with cold acetone, dried, and recrystallized from a mixture of solvents 2-propanol-hexane (2:1) or DMF:2-propanol (for **9c**).

Metod B. A mixture of ammonium thiocyanate (1.2 mmol), acetyl chloride (for compounds **10a-c**) or benzoyl chloride (for compounds **11a-c**) in 20 mL of acetone is heated with reflux and stirring for 2 hours. The resulting precipitate of KCl is filtered off and immediately added to a solution of 1 mmol of the corresponding 3-aminopyridin-2(1*H*)-one (**7a-c**) in 10 mL of acetone and stirred for an additional 3 hours. The reaction mixture with the precipitated solid is cooled, filtered, washed with acetone, and dried. After recrystallization from a mixture of 2-propanol-DMF (2:1), compounds **10-11a-c** are obtained.

The physicochemical constants and spectral characteristics of thioureas 1-allyl-3-(4,6-dimethyl-2-oxo-1,2-dihydropyridin-3-yl)thiourea (**8a**), 1-allyl-3-(6-methyl-2-oxo-4-phenyl-1,2-dihydropyridin-3-yl)thiourea (**8b**), N-((4,6-dimethyl-2-oxo-1,2-dihydropyridin-3-yl)carbamothioyl)benzamide (**11a**), and N-((6-methyl-2-oxo-4-phenyl-1,2-dihydropyridin-3-yl)carbamothioyl)benzamide (**11b**) were described by us in [31].

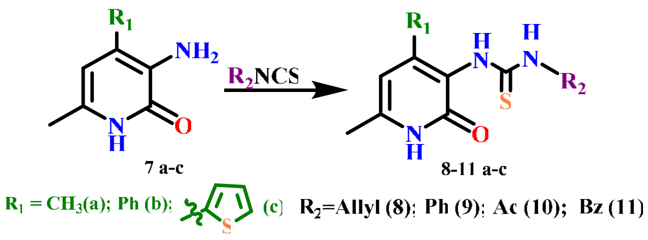

Characterization data of products

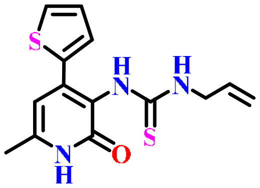<br>Chemica Formula: $C_{14}H_{15}N_3OS_2$<br>Molecular Weight: 305,41	**1-Allyl-3-(6-methyl-2-oxo-4-(thiophen-2-yl)-1,2-dihydropyridin-3-yl)thiourea (8c)**<br>Yield 360 mg (59%), M.p.: = 289-292 °C. ^{1}H NMR (400 MHz, DMSO-d6) δ ppm (*J*, Hz): 2.20 (s, 3H, 6-CH_3); 4.05 (br. s, 2H, CH_2); 4.98 (br. s., 1H, C=H_a); 5.11 (br. d, *J*=15.1 Hz, 1H, C=H_b); 5.76 (br. s., 1H, -CH=CH_aH_b); 6.45 (s, 1H, H-5); 7.14 (d, *J*=4.1 Hz, 1H, H-4 thiophene); 7.61 (br. s. 1H, NH-CH_2); 7.66 (d, *J*=3.7 Hz, 1H, H-3 thiophene); 7.71 (d, *J*=4.6 Hz, 1H, H-5 tiophene); 8.52 (br. s., 1H, NHCS); 11.64 (br. s., 1H, NHCO). ^{13}C NMR (101 MHz, DMSO-d6) δ ppm 18.5 (CH_3); 46.3 (NH-CH_2); 102.6; 114.9 (=CH_2),; 120.5; 126.9 (C-3 thiophene); 128.7 (C-4 thiophene); 130.3 (C-5 thiophene); 135.1 (CH_2C=); 137.3; 141.0; 143.3; 161.0; 189.0 (C=S).
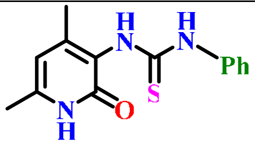<br>Chemical Formula: $C_{14}H_{15}N_3OS$<br>Molecular Weight: 273,35	**1-(4,6-dimethyl-2-oxo-1,2-dihydropyridin-3-yl)-3-phenylthiourea (9a)**<br>Yield: 0.180 g (66%), white powder, M.p.: 140-143°C. ^{1}H NMR (500 MHz, DMSO-d6) δ ppm (*J*, Hz): 2.05 (s, 1H, CH_3); 2.13 (s, 1H, CH_3); 5.90 (s, 1H, H-5); 7.09 (t, 1H, *J*=7.2 Hz, H-4 Ph); 7.30 (t, 2H, *J*=7.6 Hz, H-3,5 Ph); 7.50 (d, 2H, *J*=7.3 Hz, H-3,5 Ph); 8.68 (br. s., 1H, NHPh); 9.57 (br. s., 1H, NHCS); 11.63 (br. s., 1H, NHCO). ^{13}C NMR (125 MHz, DMSO-d6) δ ppm 18.2 (CH_3); 18.2 (CH_3); 106.6 (C-5); 124.1 (2C Ph); 125.9 (C Ph); 128.3 (2C Ph); 129.9; 139.7; 142.3; 147.9; 160.4; 180.7. HRMS m/z: calcd for $C_{14}H_{16}N_3OS^+$ [M + H]+: 274.1009; found: 274.0099.
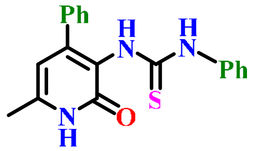<br>Chemical Formula: $C_{19}H_{17}N_3OS$<br>Molecular Weight: 335,43	**1-(6-methyl-2-oxo-4-phenyl-1,2-dihydropyridin-3-yl)-3-phenylthiourea (9b)**<br>Yield: 0.232 g (69%), white powder, M.p.: 141-144°C. ^{1}H NMR (81 MHz, DMSO-d6) δ ppm (*J*, Hz): 2.22 (s, 1H, CH_3); 6.03 (s, 1H, H-5); 7.14-7.43 (m, 10H, H-2,3,4,5,6 Ph, H-2,3,4,5,6 Ar); 8.67 (br. s., 1H, NHPh); 9.51 (br. s, 1H, NHCS); 11.83 (br. s., 1H, NHCO). ^{13}C NMR (20 MHz, DMSO-d6) δ ppm 18.4 (CH_3); 105.8 (C-5); 123.6 (2C Ph); 128.2 (8C Ph); 137.6; 139.6; 143.3; 149.0; 160.8; 162.3; 181.2. HRMS m/z: calcd for $C_{19}H_{18}N_3OS^+$ $[M + H]^+$: 336.1165; found: 336.1175.

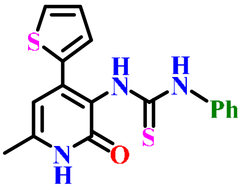<br>Chemica Formula: $C_{17}H_{15}N_3OS_2$<br>Molecular Weight: 341,45	**1-(6-methyl-2-oxo-4-(thiophen-2-yl)-1,2-dihydropyridin-3-yl)-3-phenylthiourea (9c)**<br>Yield: 0.260 g (76%), white powder, M.p.: 145-147°C. ^{1}H NMR (81 MHz, DMSO-d6) δ ppm (*J*, Hz): 2.22 (s, 1H, CH_3); 6.47 (s, 1H, H-5); 7.09-7.76 (m, 8H, H-2,3,4,5,6 Ph, H-3,4,5 thiophene); 8.76 (s, 1H, NHPh); 9.73 (br.s, 1H, NHCS); 11.69 (br. s., 1H, NHCO). ^{13}C NMR (20 MHz, DMSO-d6) δ ppm 18.5 (CH_3); 102.8 (C-5); 124.3 (2C Ph); 126.9 (1C thiophene); 128.2 (5C Ph); 128.7 (1C thiophene); 130.2 (1C thiophene); 137.4; 139.6; 141.4; 143.0; 160.9; 162.3; 181.6. HRMS m/z: calcd for $C_{17}H_{16}N_3OS_2^+$ [M + H]+: 342.0729; found: 342.0735.
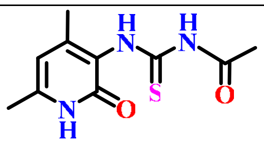<br>Chemica Formula: $C_{10}H_{13}N_3O_2S$<br>Molecular Weight: 239,29	**N-((4,6-dimethyl-2-oxo-1,2-dihydropyridin-3-yl)carbamothioyl)acetamide (10a)**<br>Yield: 0.127 g (53%), light beige powder, M.p.: 273-275°C. ^{1}H NMR (500 MHz, DMSO-d6) δ ppm (*J*, Hz): 1.99 (s, 3H, CH_3); 2.12 (s, 6H, 2CH_3); 5.90 (s, 1H, H-5); 11.42 (br. s., 1H, NHCS); 11.44 (br. s., 1H, NHCO); 11.69 (br. s., 1H, NHCO). ^{13}C NMR (125 MHz, DMSO-d6) δ ppm 18.18 (CH_3); 18.21 (CH_3); 23.7 (CH_3); 106.6 (C-5); 123.1; 143.0; 147.6; 159.5; 172.4; 180.7. HRMS m/z: calcd for $C_{10}H_{14}N_3O_2S^+$ [M + H]+: 240.0801; found: 240.0810.
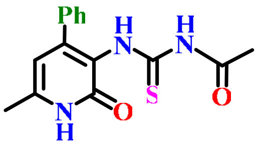<br>Chemica Formula: $C_{15}H_{15}N_3O_2S$<br>Molecular Weight: 301,36	**N-((6-methyl-2-oxo-4-phenyl-1,2-dihydropyridin-3-yl)carbamothioyl)acetamide (10b)**<br>Yield: 0.168 g (56%), light beige powder, M.p.: 249-251°C. ^{1}H NMR (500 MHz, DMSO-d6) δ ppm (*J*, Hz): 2.05 (s, 3H, CH_3); 2.21 (s, 3H, CH_3); 6.02 (s, 1H, H-5); 7.35-7.39 (m, 3H, H-3,4,5 Ph); 7.42-7.45 (m, 2H, H-2,6 Ph); 11.29 (br. s., 1H, NHCS); 11.34 (br. s., 1H, NHCO); 11.91 (br. s, 1H, NHCO). ^{13}C NMR (125 MHz, DMSO-d6) δ ppm 18.4 (CH_3); 23.6 (CH_3); 105.5 (C-5); 122.1; 127.6 (2C Ph); 128.2 (2C Ph); 128.5; 137.1; 144.0; 148.9; 159.5; 172.2; 181.4. HRMS m/z: calcd for $C_{15}H_{16}N_3O_2S^+$ $[M + H]^+$: 302.0958; found: 302.0963.

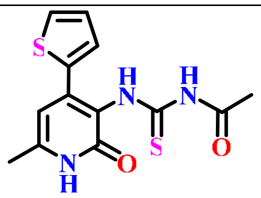<br>Chemical Formula: $C_{13}H_{13}N_3O_2S_2$<br>Molecular Weight 307,39	**N-((6-methyl-2-oxo-4-(thiophen-2-yl)-1,2-dihydropyridin-3-yl)carbamothioyl)acetamide (10c)**<br>Yield: 0.169 g (55%), light beige powder, M.p.: 252-254°C. ^{1}H NMR (500 MHz, DMSO-d6) δ ppm (*J*, Hz): 2.16 (s 3H, CH$_3$'); 2.20 (s, 3H, CH$_3$); 6.46 (s, 1H, H-5); 7.14 (dd, 1H, *J*=5.0 Hz, *J*=3.7 Hz, H-4 thiophene); 7.64 (dd, 1H, *J*=3.8 Hz, *J*=1.1 Hz, H-3 thiophene); 7.72 (dd, 1H, *J*=5.1 Hz, *J*=1.0 Hz, H-5 tiophene); 11.47 (br. s, 1H, NHCS); 11.56 (br. s, 1H, NHCO); 11.77 (br. s, 1H, NHCO). ^{13}C NMR (125 MHz, DMSO-d6) δ ppm 18.5 (CH$_3$); 23.7 (CH$_3$); 102.6 (C-5); 120.2; 127.2 (C-3 thiophene); 129.00 (C-4 thiophene); 130.3 (C-5 thiophene); 136.8; 140.6; 143.6; 159.7; 172.5; 182.2. HRMS m/z: calcd for $C_{13}H_{14}N_3O_2S_2^+$ $[M+H]^+$: 308.0522; found: 308.0530.
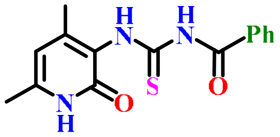<br>Chemical Formula $C_{15}H_{15}N_3O_2S$<br>Molecular Weight: 301,3640	**N-[(4,6-Dimethyl-2-oxo-1,2-dihydropyridin-3-yl)carbamothioyl]benzamide (11a)**<br>Yield: 0.682 g (76%), light-yellow, finely crystalline powder, M.p.: 230-232°C. ^{1}H NMR (400 MHz, DMSO-d6) δ ppm (*J*, Hz): 2.07 (s, 3H, 4-CH$_3$); 2.16 (s, 3H, 6-CH$_3$); 5.92 (s, 1H, H-5); 7.53 (t, *J*=7.6 Hz, 2H, H-3,5 Ph); 7.65 (t, *J*=7.6 Hz, 1H, H-4 Ph); 7.98 (d, *J*=7.8 Hz, 2H, H-2,6 Ph); 11.42 (br. s, 1H, NHCS); 11.62 (br. s, 1H, NHCO); 11.64 (br. s, 1H, NHCO). ^{13}C NMR (100 MHz, DMSO-d6) δ ppm 18.2 (4,6-CH$_3$); 106.6 (C-5); 123.4 (C-4); 128.4 (C-2,6 Ph); 128.6 (C-3,5 Ph); 132.1 (C-1 Ph); 133.1 (C-4 Ph); 143.1 (C-3); 147.6 (C-6); 159.5 (C-2); 168.3 (CO); 180.9 (CS). HRMS m/z: calcd for $C_{15}H_{16}N_3O_2S^+$ $[M + H]^+$: 302.0958; found: 302.0963.
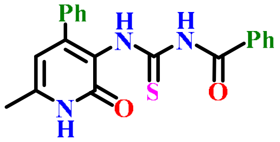<br>Chemical Formula $C_{20}H_{17}N_3O_2S$<br>Molecular Weight: 363,4350	**N-[(6-Methyl-2-oxo-4-phenyl-1,2-dihydropyridin-3-yl)carbamothioyl]benzamide (11b)**<br>Yield: 0.244 g (67%), light-yellow, finely crystalline powder, M.p.: 218-220°C. ^{1}H NMR (400 MHz, DMSO-d6) δ ppm (*J*, Hz): 2.24 (s, 3H, 6-CH$_3$); 6.05 (s, 1H, H-5); 7.34-7.41 (m, 3H, H-2,4,6 Ph); 7.47-7.51 (m, 4H, H-3,5 Ph, H-3,5 Ph'); 7.62 (t, *J*=7.4 Hz, 1H, H-4 Ph'); 7.91 (d, *J*=7.2 Hz, 2H, H-2,6 Ph'); 11.41 (br. s, 1H, NHCS); 11.63 (br. s, 1H, NHCO); 11.92 (br. s, 1H, NHCO). ^{13}C NMR (100 MHz, DMSO-d6) δ ppm 18.9 (6-CH$_3$); 106.0 (C-5); 122.8 (C-1 Ph); 128.1 (C-2,6 Ph); 128.7

	(C-3,5 Ph); 128.9 (C-4 Ph, C-2,6 Bz); 129.0 (C-3,5 Bz); 132.4 (C-1 Bz); 133.6 (C-4 Bz); 137.6 (C-4); 144.5 (C-3); 149.4 (C-6); 160.0 (C-2); 168.4 (CO); 182.0 (CS). HRMS m/z: calcd for $C_{20}H_{18}N_3O_2S^+$ $[M + H]^+$: 364.1114; found: 364.1113.
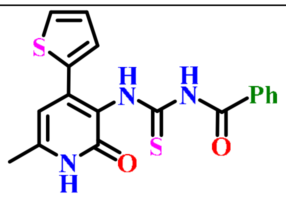<br>Chemical Formula: $C_{18}H_{15}N_3O_2S_2$<br>Molecular Weight 369,46	**N-((6-methyl-2-oxo-4-(thiophen-2-yl)-1,2-dihydropyridin-3-yl)carbamothioyl)benzamide (11c)**<br>Yield: 0.314 g (85%), light beige powder, M.p.: 245-249°C. ^{1}H NMR (500 MHz, DMSO-d6) δ ppm (*J*, Hz): 2.23 (s, 3H, CH_3); 6.50 (s, 1H, H-5); 7.15 (dd, 1H, *J*=4.9 Hz, *J*=4.1 Hz, H-4 thiophene); 7.55 (t, 2H, H-3,5 Ph); 7.66 (m, 1H, H-4 Ph); 7.69 (d, 1H, H-3 thiophene); 7.72 (d, 1H, H-5 thiophene); 8.01 (d, 2H, H-2,6 Ph); 11.76 (br. s, 2H, NHCS, NHCO); 11.81 (br. s, 1H, NHCO). ^{13}C NMR (125 MHz, DMSO-d6) δ ppm 18.5 (CH_3); 102.7 (C-5); 120.4; 127.2; 128.5 (2C Ph); 128.7 (2C Ph); 129.0; 130.2; 132.0; 133.2; 136.8; 140.6; 143.6; 159.7; 168.2; 182.4. HRMS m/z: calcd for $C_{18}H_{15}N_3O_2S_2^+$ $[M + H]^+$: 370.0678; found: 370.0670.

Author Contributions

Spectrophotometric studies were performed on the basis of the Research Resource Center “Natural Resource Management and Physico-Chemical Research” Institute of Chemistry, Tyumen State University.

Copies of NMR Spectra of Products

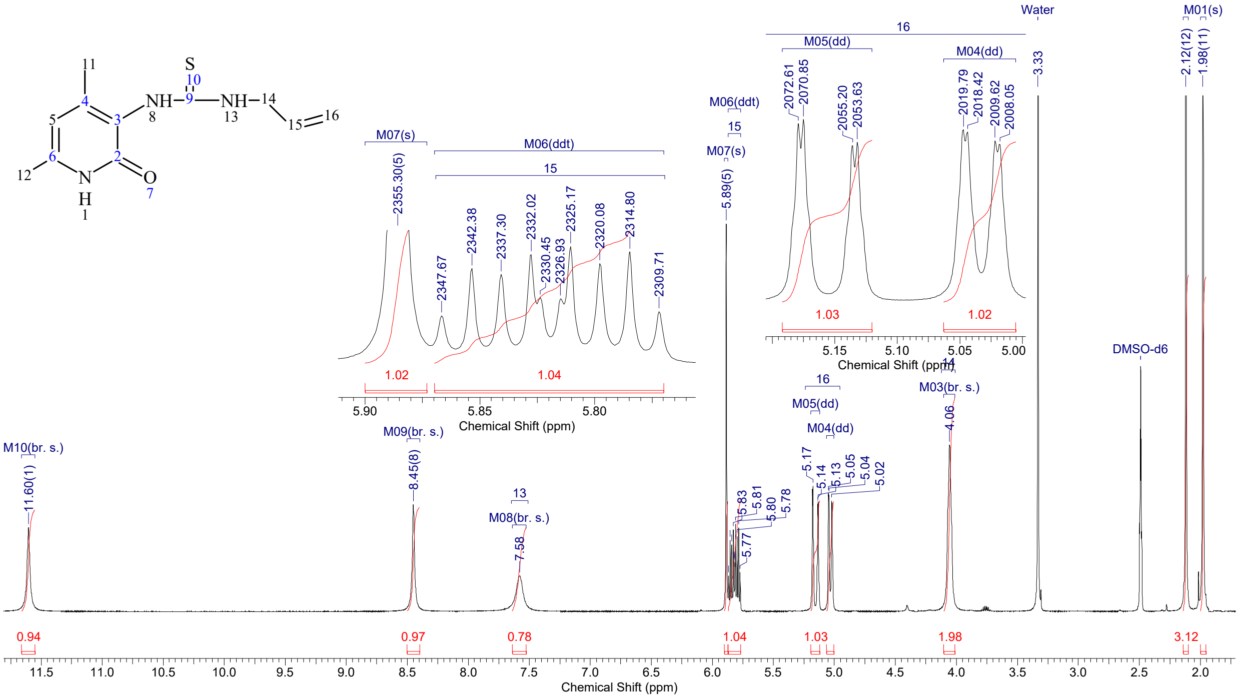

No.	(ppm)	(Hz)	Height	No.	(ppm)	(Hz)	Height
1	18.04	1815.6	-0.3050	6	135.09	13593.1	-0.1314
2	18.14	1825.1	-0.4010	7	142.34	14322.3	0.0826
3	46.41	4670.1	0.2443	8	148.24	14916.5	0.1430
4	106.80	10746.6	-0.3011	9	160.46	16146.1	0.2636
5	115.22	11594.0	0.2606	10	181.90	18303.6	0.0686

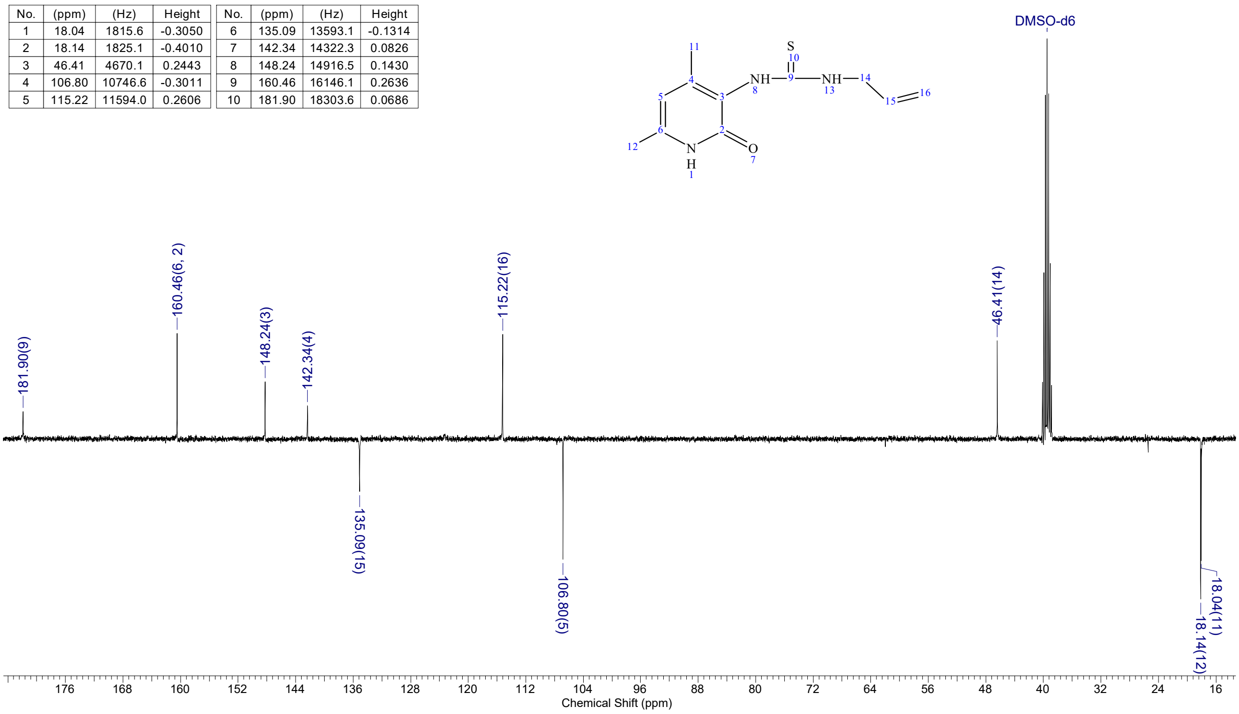

^{1}H (400 MHz, DMSO-d6) and ^{13}C (100 MHz, DMSO-d6) NMR Spectra of **8a**

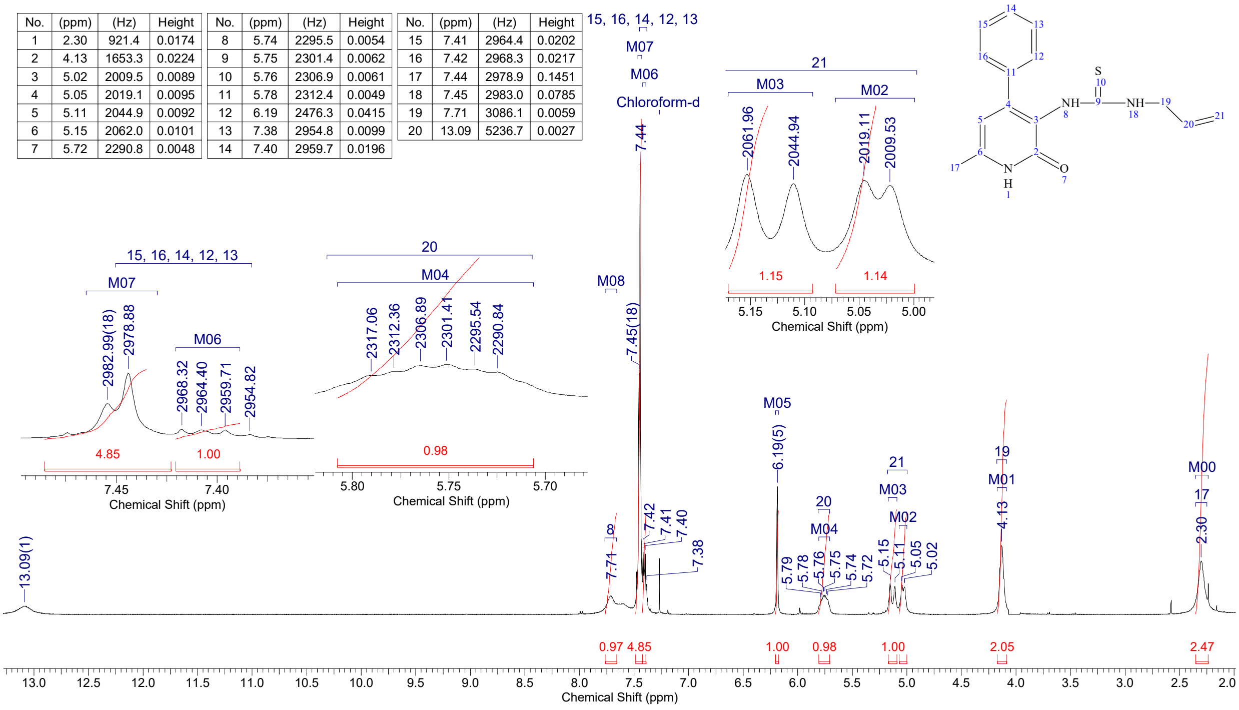

No.	(ppm)	(Hz)	Height
1	2.30	921.4	0.0174
2	4.13	1653.3	0.0224
3	5.02	2009.5	0.0089
4	5.05	2019.1	0.0095
5	5.11	2044.9	0.0092
6	5.15	2062.0	0.0101
7	5.72	2290.8	0.0048
8	5.74	2295.5	0.0054
9	5.75	2301.4	0.0062
10	5.76	2306.9	0.0061
11	5.78	2312.4	0.0049
12	6.19	2476.3	0.0415
13	7.38	2954.8	0.0099
14	7.40	2959.7	0.0196
15	7.41	2964.4	0.0202
16	7.42	2968.3	0.0217
17	7.44	2978.9	0.1451
18	7.45	2983.0	0.0785
19	7.71	3086.1	0.0059
20	13.09	5236.7	0.0027

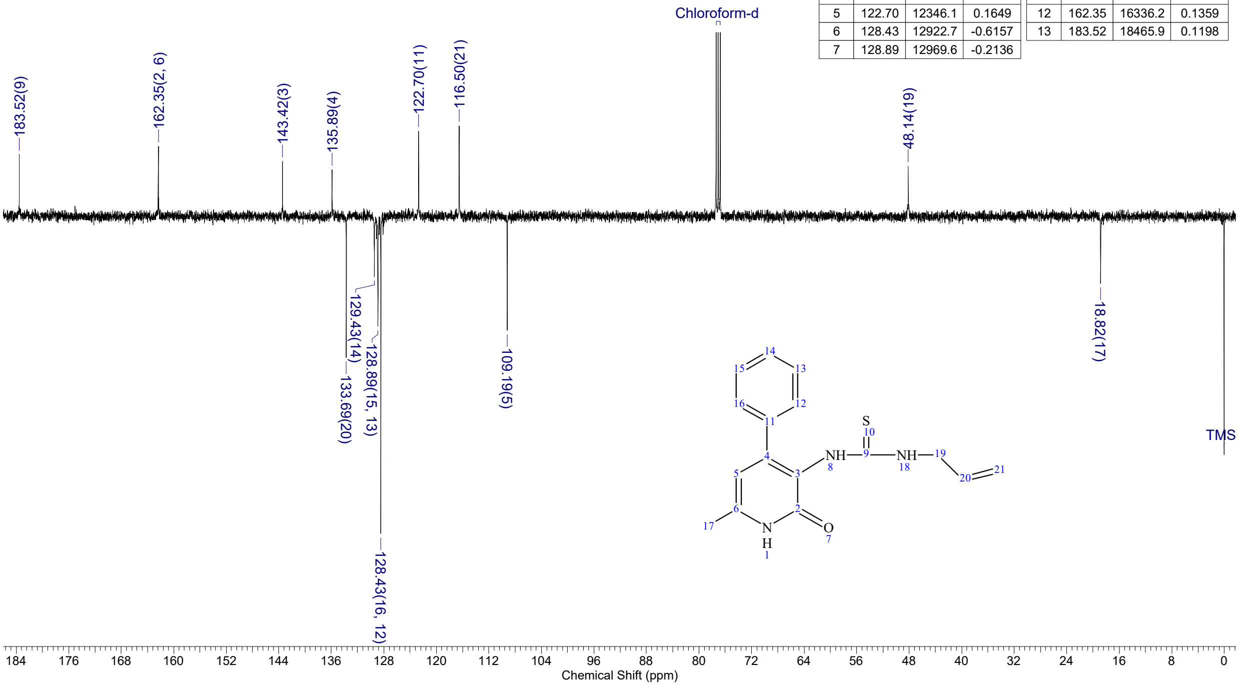

No.	(ppm)	(Hz)	Height
1	18.82	1894.2	-0.1305
2	48.14	4844.1	0.0970
3	109.19	10987.4	-0.2219
4	116.50	11722.5	0.1755
5	122.70	12346.1	0.1649
6	128.43	12922.7	-0.6157
7	128.89	12969.6	-0.2136
8	129.43	13023.9	-0.1166
9	133.69	13452.4	-0.2743
10	135.89	13673.2	0.0905
11	143.42	14431.7	0.1063
12	162.35	16336.2	0.1359
13	183.52	18465.9	0.1198

^{1}H (400 MHz, $CDCl_3$) and ^{13}C (100 MHz, $CDCl_3$) NMR Spectra of **8b**

No.	(ppm)	(Hz)	Height
1	2.20	880.1	1.0000
2	2.49	995.5	0.8527
3	4.05	1621.0	0.0952
4	4.98	1992.4	0.0293
5	5.09	2035.0	0.0206
6	5.13	2050.1	0.0216

No.	(ppm)	(Hz)	Height
7	5.76	2303.8	0.0209
8	6.45	2579.5	0.2181
9	7.13	2852.0	0.1717
10	7.14	2856.1	0.1753
11	7.61	3040.7	0.0675
12	7.66	3064.0	0.1437

No.	(ppm)	(Hz)	Height
13	7.67	3067.7	0.1367
14	7.71	3083.2	0.1632
15	7.72	3087.8	0.1563
16	8.52	3405.6	0.0404
17	11.64	4654.0	0.0484

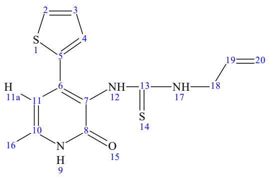

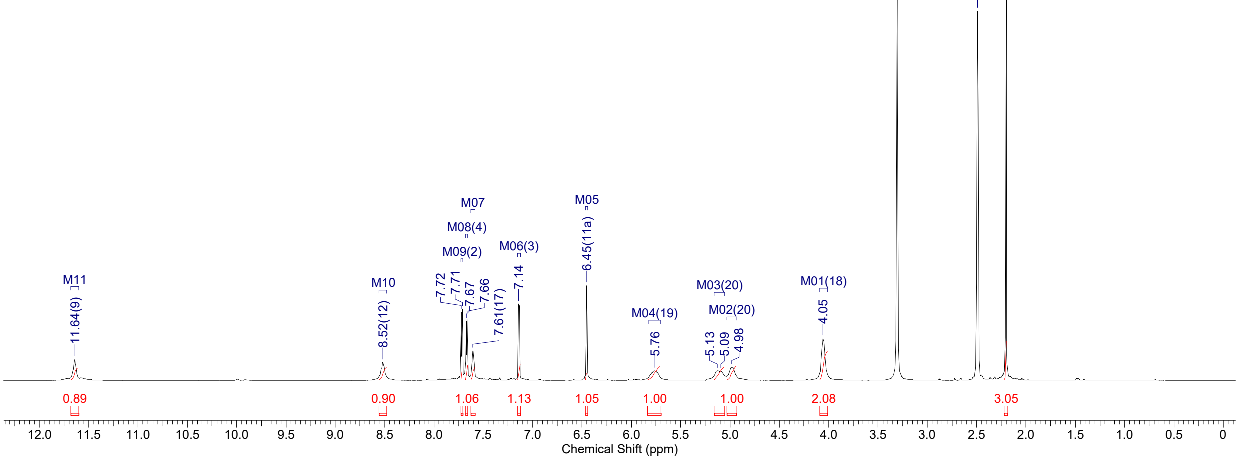

No.	(ppm)	(Hz)	Height
1	18.47	1856.4	0.0460
2	39.50	3970.7	1.0000
3	46.34	4658.0	0.0294
4	102.57	10311.0	0.0198
5	114.86	11546.5	0.0120
6	120.46	12109.1	0.0120
7	126.92	12758.9	0.0254
8	128.71	12938.2	0.0266

No.	(ppm)	(Hz)	Height
9	130.33	13101.1	0.0218
10	135.07	13578.4	0.0135
11	137.26	13797.9	0.0102
12	140.98	14171.7	0.0105
13	143.28	14403.7	0.0100
14	160.98	16182.6	0.0124
15	188.97	18996.6	0.0057

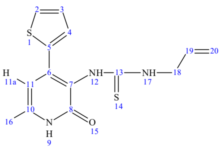

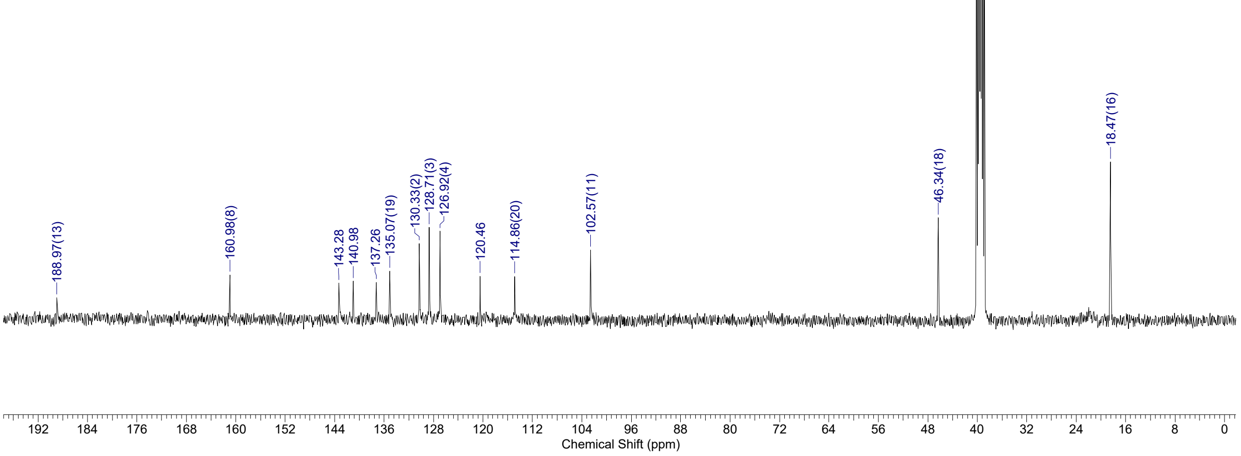

^{1}H (400 MHz,$CDCl_3$) and ^{13}C (100 MHz, $CDCl_3$) NMR Spectra of **8c**

No.	(ppm)	(Hz)	Height
1	2.05	1022.8	0.6512
2	2.13	1066.1	0.6350
3	5.90	2953.2	0.2020
4	7.07	3538.4	0.0548
5	7.09	3545.7	0.1127
6	7.10	3552.8	0.0713
7	7.28	3643.1	0.1207

No.	(ppm)	(Hz)	Height
8	7.30	3650.6	0.2186
9	7.31	3658.2	0.1301
10	7.50	3749.4	0.1666
11	7.51	3756.8	0.1483
12	8.68	4342.4	0.0651
13	9.67	4834.9	0.0257
14	11.63	5816.3	0.1017

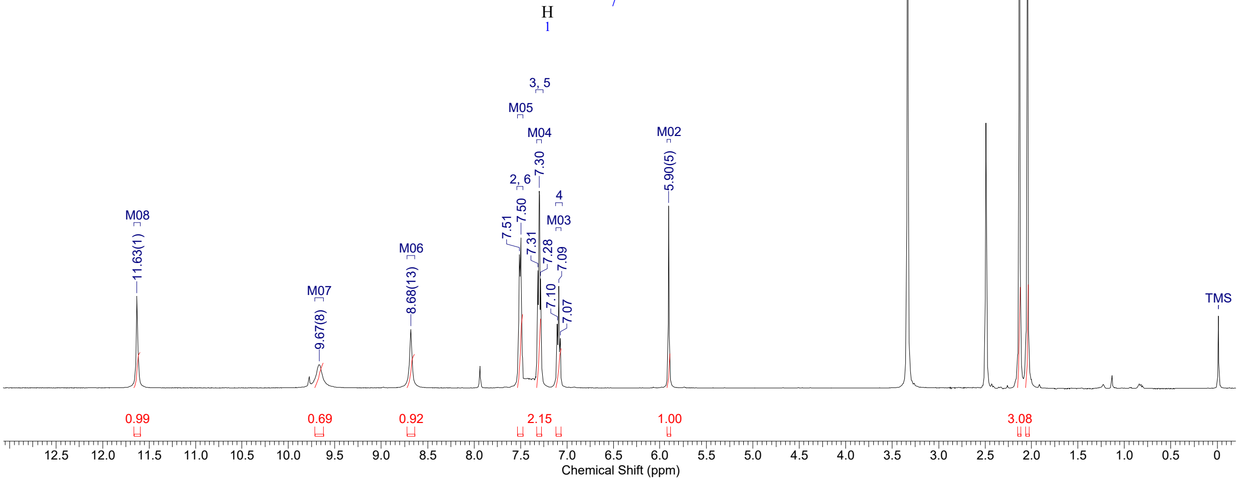

No.	(ppm)	(Hz)	Height
1	18.16	2283.9	0.1518
2	18.19	2288.1	0.1433
3	106.82	13433.0	0.0931
4	124.13	15609.9	0.1145
5	125.89	15831.2	0.0513
6	128.27	16130.8	0.1884

No.	(ppm)	(Hz)	Height
7	129.87	16332.8	0.0645
8	139.66	17563.1	0.0428
9	142.30	17895.4	0.0316
10	147.95	18605.7	0.0662
11	160.38	20169.2	0.0665
12	180.74	22729.9	0.0480

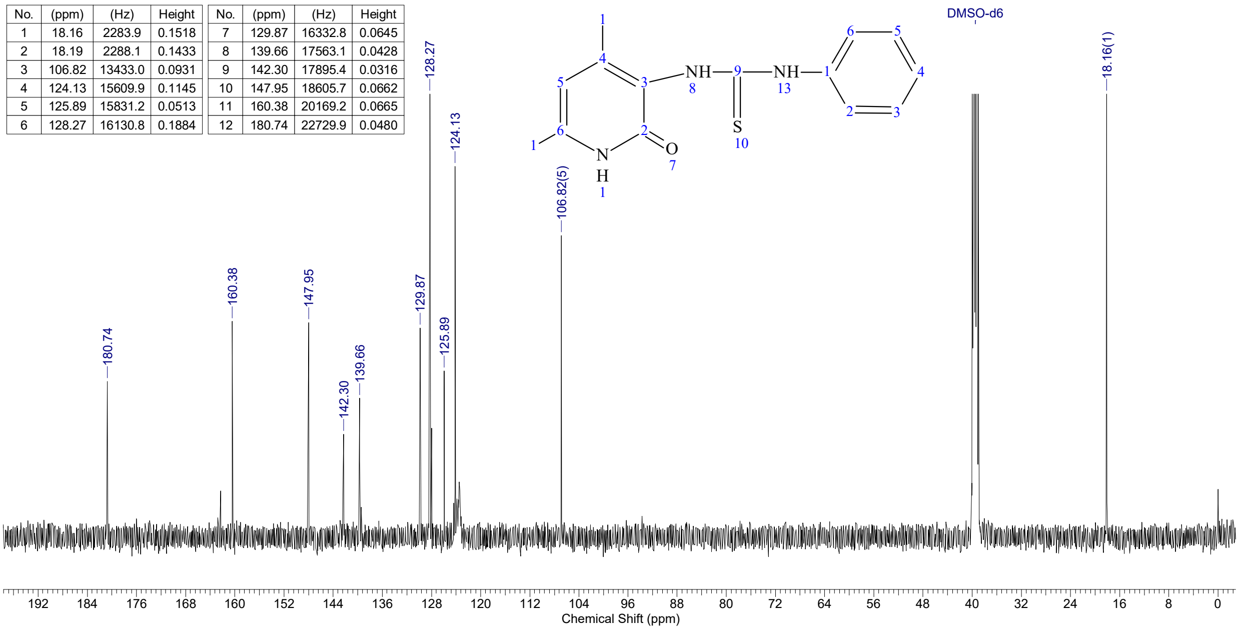

^{1}H (500 MHz, DMSO-d6) and ^{13}C (125 MHz, DMSO-d6) NMR Spectra of **9a**

No.	(ppm)	(Hz)	Height
1	2.22	178.7	1.0000
2	6.03	485.0	0.3874
3	7.14	575.0	0.2476
4	7.31	588.6	0.8306
5	7.43	598.2	0.8437
6	8.67	698.3	0.3214
7	9.51	765.4	0.2764
8	11.83	952.4	0.1377

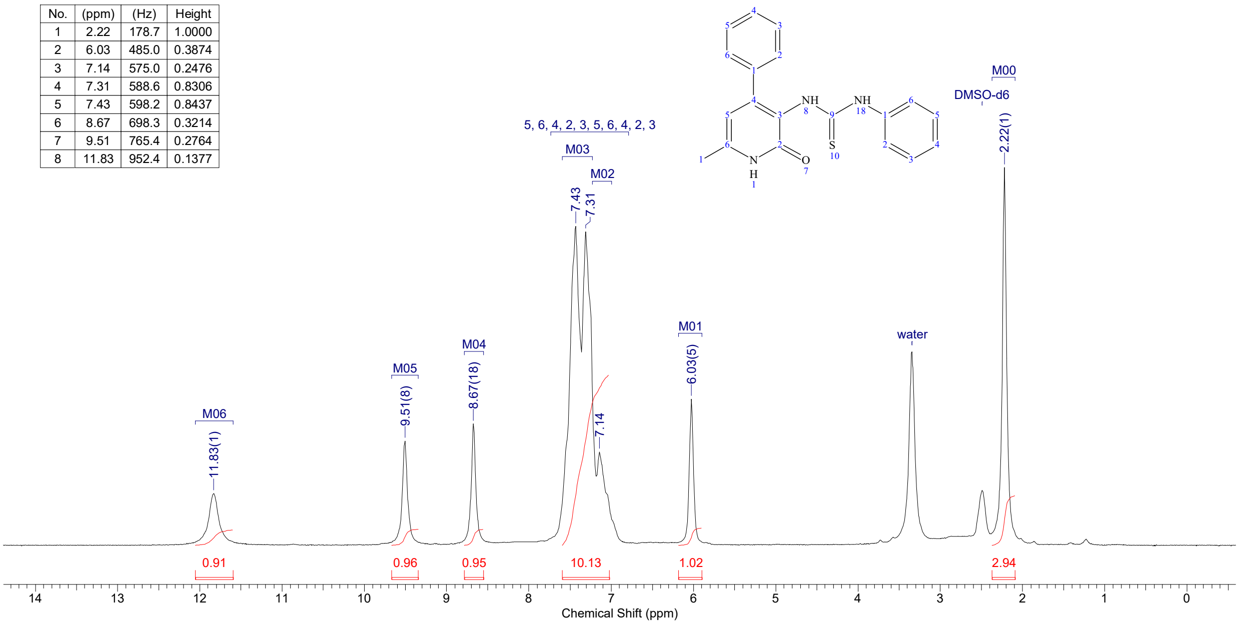

No.	(ppm)	(Hz)	Height
1	18.42	372.8	0.2667
2	105.81	2142.1	0.2137
3	123.62	2502.6	0.3889
4	128.19	2595.1	1.0000
5	137.58	2785.1	0.2056
6	139.59	2825.9	0.1995

No.	(ppm)	(Hz)	Height
7	143.33	2901.6	0.2372
8	149.02	3016.7	0.1869
9	160.78	3254.8	0.2380
10	162.27	3285.0	0.1590
11	181.17	3667.6	0.2065

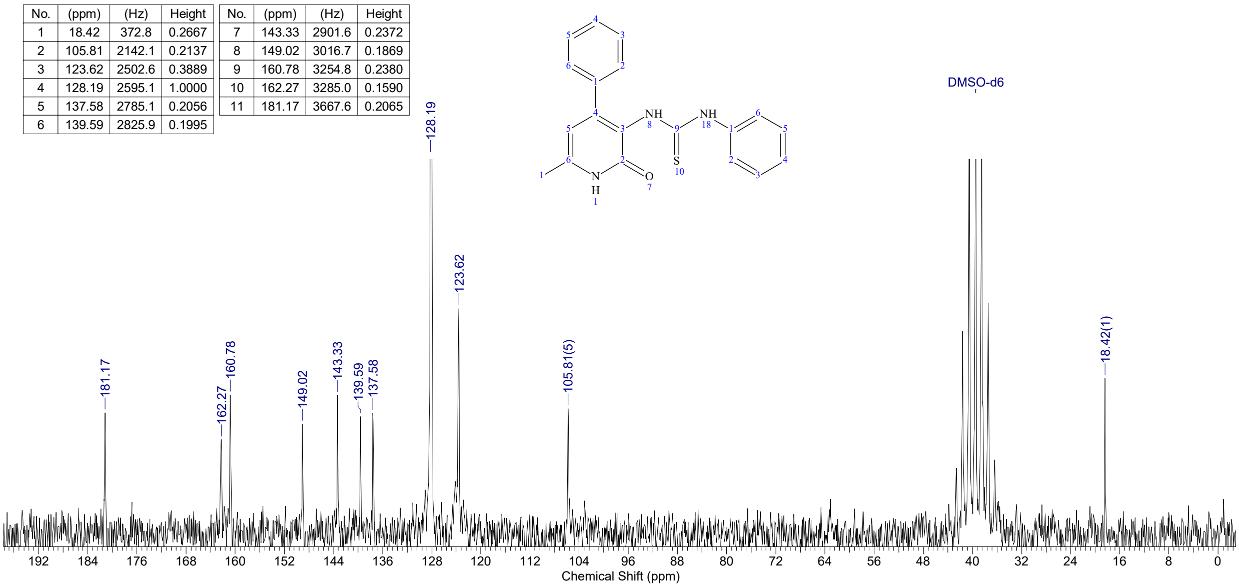

^{1}H (81 MHz, DMSO-d6) and ^{13}C (20 MHz, DMSO-d6) NMR Spectra of **9b**

No.	(ppm)	(Hz)	Height	No.	(ppm)	(Hz)	Height
1	2.22	178.4	1.0000	8	7.47	601.0	0.3793
2	6.47	520.7	0.3743	9	7.55	608.1	0.1980
3	7.09	570.9	0.2448	10	7.70	620.0	0.4628
4	7.15	575.9	0.4455	11	7.76	624.5	0.2527
5	7.20	579.3	0.4285	12	8.76	705.1	0.2311
6	7.29	587.1	0.3885	13	9.73	783.6	0.0849
7	7.38	594.2	0.2416	14	11.69	940.7	0.1486

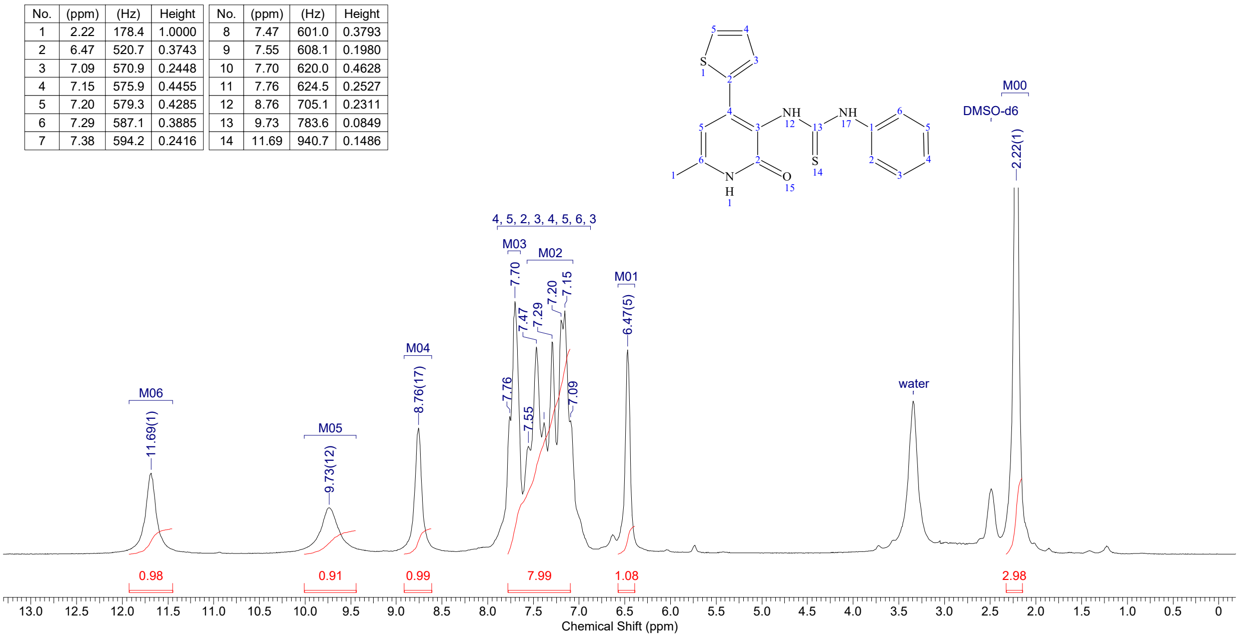

No.	(ppm)	(Hz)	Height	No.	(ppm)	(Hz)	Height
1	18.50	374.5	0.3037	8	137.38	2781.0	0.2035
2	102.77	2080.6	0.2053	9	139.66	2827.3	0.2227
3	124.29	2516.0	0.2732	10	141.37	2861.8	0.2208
4	126.97	2570.4	0.2827	11	143.02	2895.3	0.1999
5	128.20	2595.2	0.5288	12	160.98	3258.9	0.2357
6	128.71	2605.6	0.3047	13	162.27	3285.0	0.1352
7	130.24	2636.5	0.2630	14	181.65	3677.3	0.1782

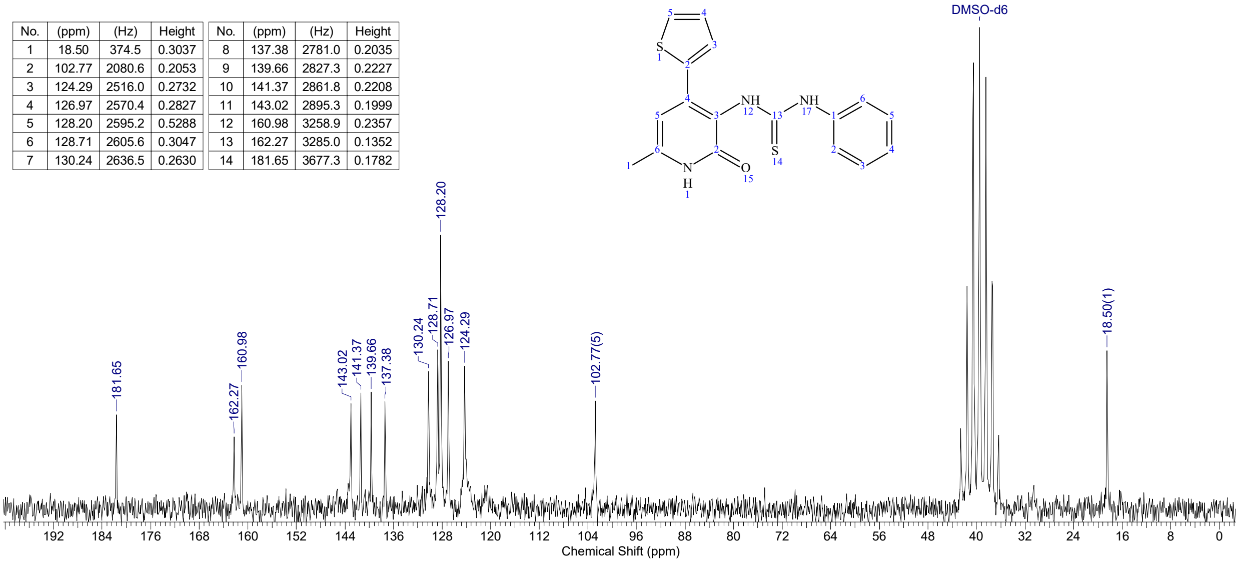

^{1}H (81 MHz, DMSO-d6) and ^{13}C (20 MHz, DMSO-d6) NMR Spectra of **9c**

No.	(ppm)	(Hz)	Height
1	1.99	997.5	0.7231
2	2.12	1062.1	1.0000
3	5.90	2952.9	0.2254
4	11.42	5711.4	0.1852
5	11.44	5719.4	0.1768
6	11.72	5860.5	0.0100

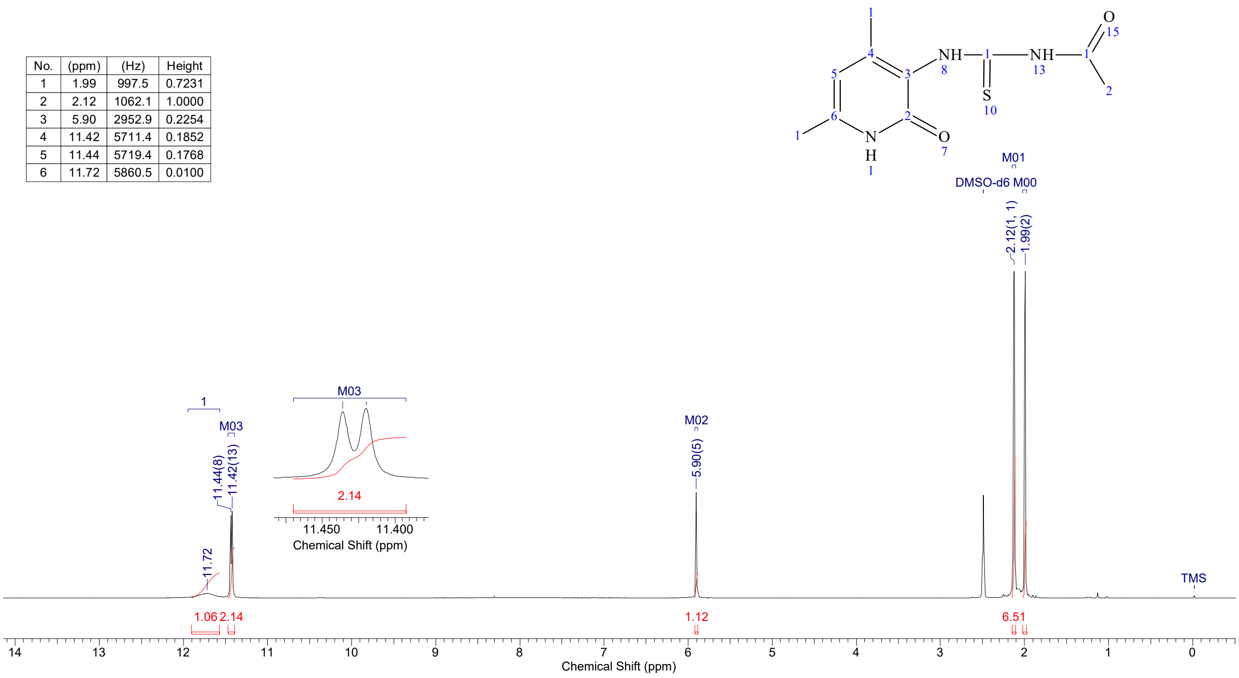

No.	(ppm)	(Hz)	Height	No.	(ppm)	(Hz)	Height
1	18.18	2286.3	0.1841	6	143.02	17986.4	0.0875
2	18.21	2290.1	0.1630	7	147.58	18559.0	0.0812
3	23.70	2980.6	0.1576	8	159.46	20053.3	0.0869
4	106.59	13404.7	0.1244	9	172.43	21684.8	0.1140
5	123.14	15485.5	0.0810	10	180.68	22721.3	0.0905

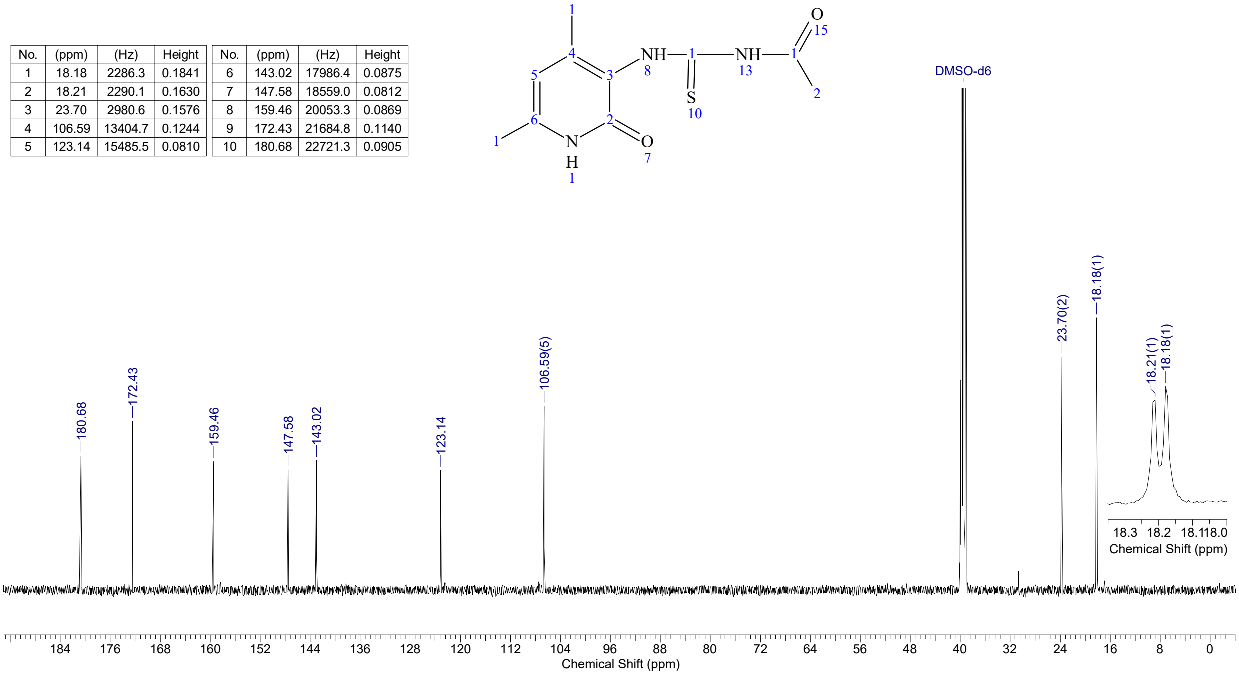

^{1}H (500 MHz, DMSO-d6) and ^{13}C (125 MHz, DMSO-d6) NMR Spectra of **10a**

No.	(ppm)	(Hz)	Height	No.	(ppm)	(Hz)	Height	No.	(ppm)	(Hz)	Height
1	2.05	1024.3	1.0000	8	7.36	3683.4	0.2021	15	7.44	3718.8	0.1135
2	2.21	1105.4	0.7507	9	7.38	3691.2	0.2473	16	7.45	3726.9	0.0343
3	2.49	1245.3	0.2279	10	7.39	3697.6	0.0968	17	11.29	5644.3	0.2096
4	6.02	3009.4	0.2465	11	7.40	3699.5	0.0760	18	11.34	5673.2	0.2185
5	7.33	3667.2	0.0318	12	7.42	3709.3	0.2403	19	11.91	5957.1	0.0427
6	7.35	3674.3	0.0942	13	7.42	3710.5	0.2455				
7	7.36	3681.2	0.1559	14	7.43	3717.1	0.1432				

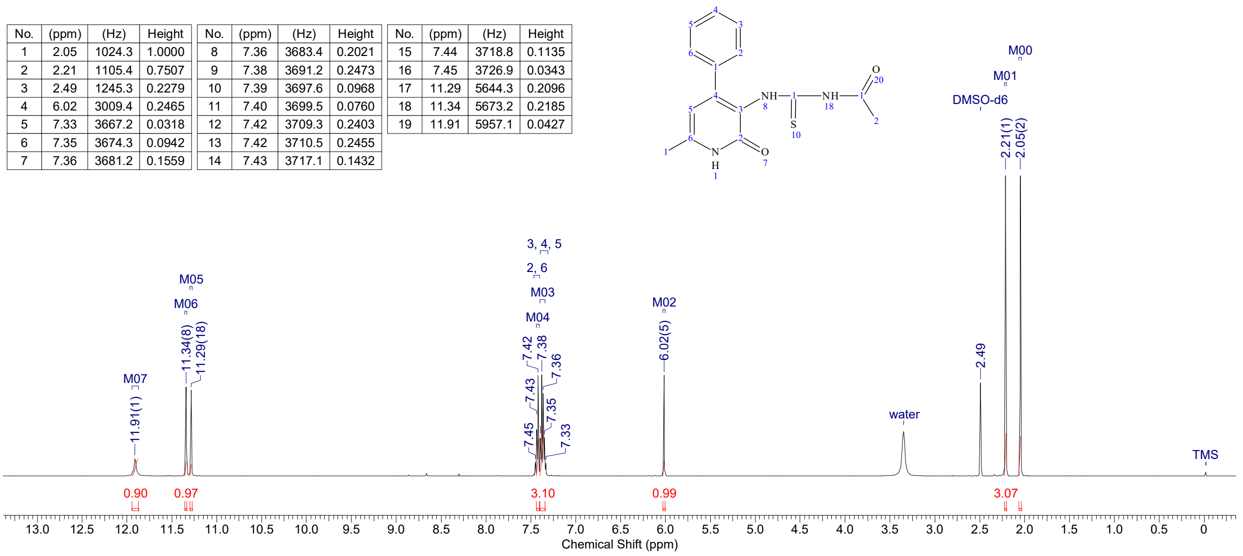

No.	(ppm)	(Hz)	Height	No.	(ppm)	(Hz)	Height
1	18.44	2318.4	0.0990	8	128.45	16153.5	0.0732
2	23.59	2967.2	0.0988	9	137.11	17243.3	0.0560
3	39.50	4967.4	1.0000	10	144.04	18114.2	0.0347
4	105.54	13272.2	0.0540	11	148.89	18723.5	0.0607
5	122.05	15349.1	0.0513	12	159.51	20059.8	0.0656
6	127.61	16048.3	0.2236	13	172.23	21658.6	0.0770
7	128.20	16122.4	0.2237	14	181.35	22805.6	0.0571

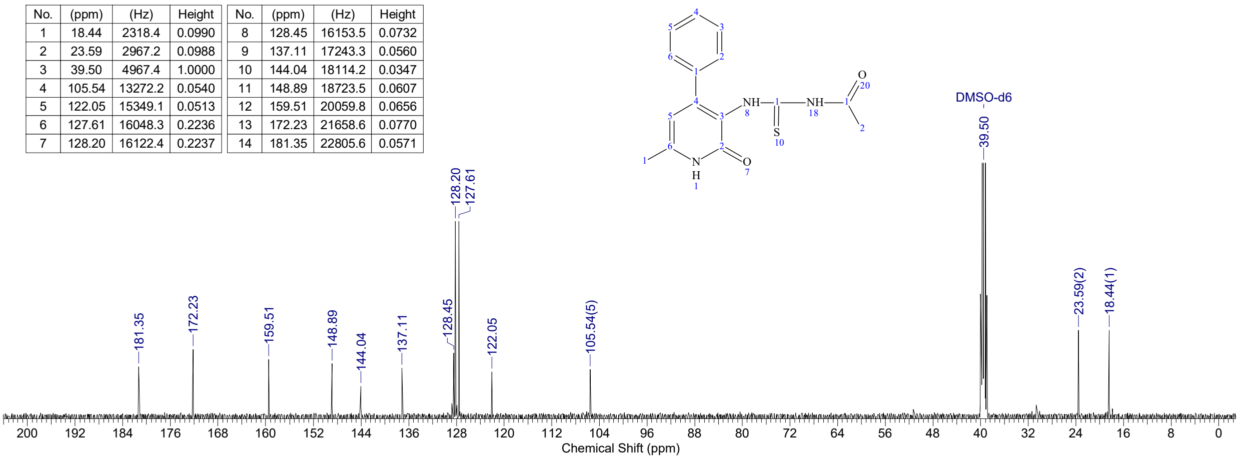

^{1}H (500 MHz, DMSO-d6) and ^{13}C (125 MHz, DMSO-d6) NMR Spectra of **10b**

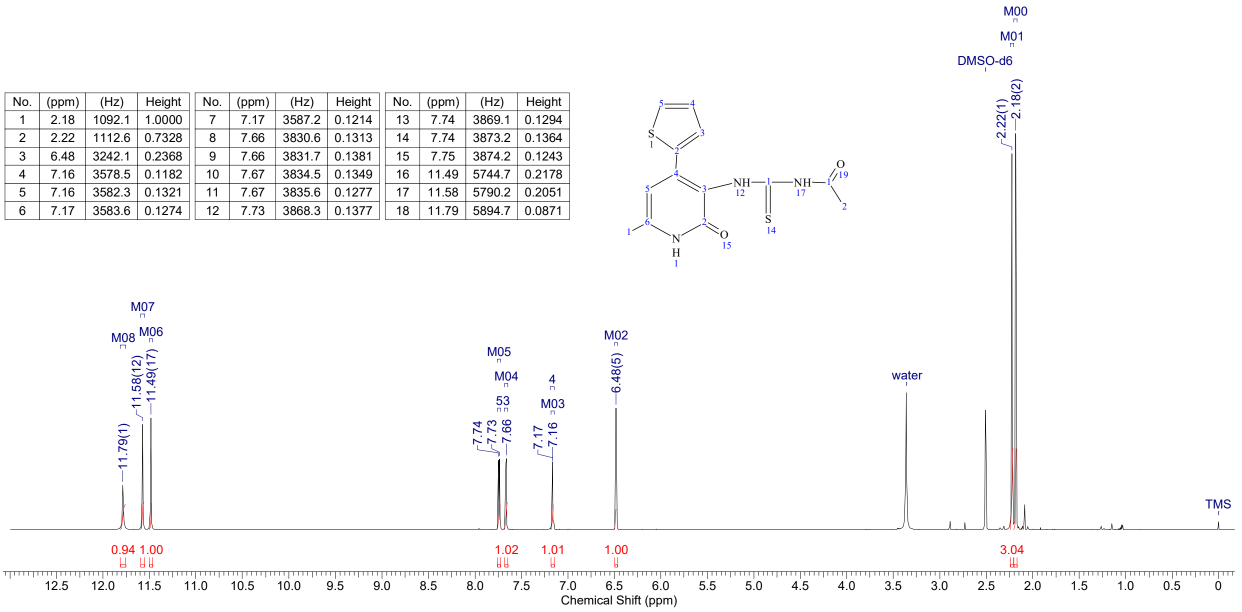

No.	(ppm)	(Hz)	Height	No.	(ppm)	(Hz)	Height	No.	(ppm)	(Hz)	Height
1	2.18	1092.1	1.0000	7	7.17	3587.2	0.1214	13	7.74	3869.1	0.1294
2	2.22	1112.6	0.7328	8	7.66	3830.6	0.1313	14	7.74	3873.2	0.1364
3	6.48	3242.1	0.2368	9	7.66	3831.7	0.1381	15	7.75	3874.2	0.1243
4	7.16	3578.5	0.1182	10	7.67	3834.5	0.1349	16	11.49	5744.7	0.2178
5	7.16	3582.3	0.1321	11	7.67	3835.6	0.1277	17	11.58	5790.2	0.2051
6	7.17	3583.6	0.1274	12	7.73	3868.3	0.1377	18	11.79	5894.7	0.0871

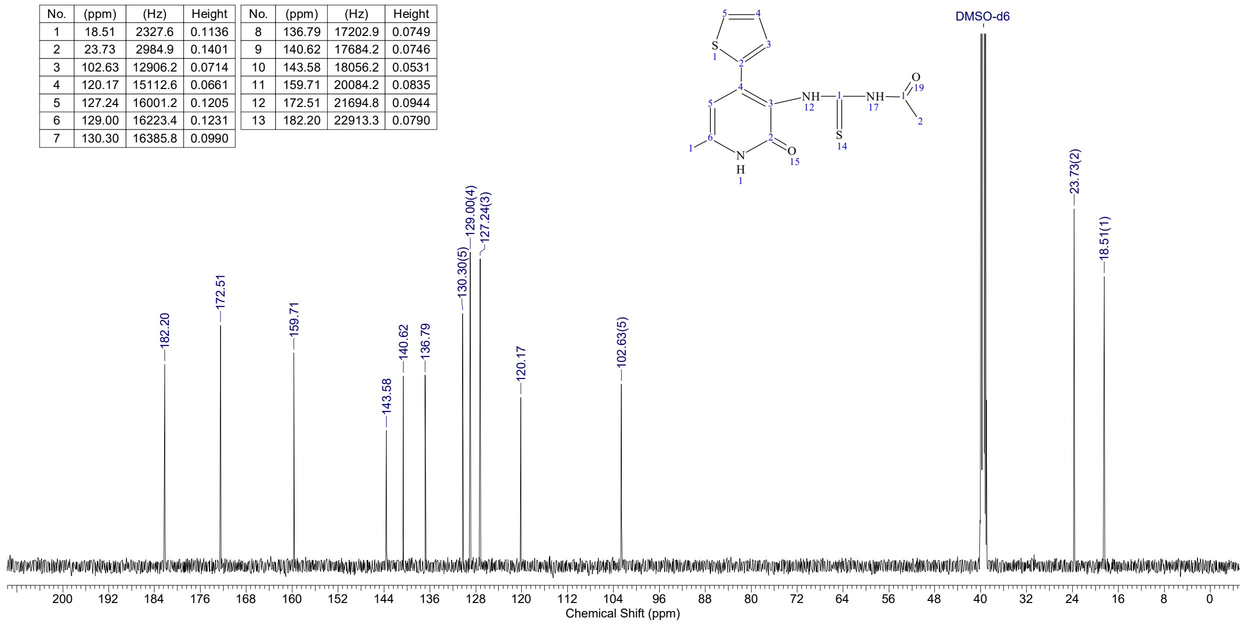

No.	(ppm)	(Hz)	Height	No.	(ppm)	(Hz)	Height
1	18.51	2327.6	0.1136	8	136.79	17202.9	0.0749
2	23.73	2984.9	0.1401	9	140.62	17684.2	0.0746
3	102.63	12906.2	0.0714	10	143.58	18056.2	0.0531
4	120.17	15112.6	0.0661	11	159.71	20084.2	0.0835
5	127.24	16001.2	0.1205	12	172.51	21694.8	0.0944
6	129.00	16223.4	0.1231	13	182.20	22913.3	0.0790
7	130.30	16385.8	0.0990				

^{1}H (500 MHz, DMSO-d6) and ^{13}C (125 MHz, DMSO-d6) NMR Spectra of **10c**

No.	(ppm)	(Hz)	Height	No.	(ppm)	(Hz)	Height
1	2.07	827.8	1.0000	8	7.63	3052.7	0.0999
2	2.16	865.0	0.9273	9	7.65	3060.4	0.1452
3	2.49	996.4	0.2054	10	7.97	3190.1	0.2861
4	5.92	2369.6	0.3112	11	7.99	3197.9	0.2687
5	7.51	3005.0	0.1331	12	11.42	4569.7	0.1495
6	7.53	3012.6	0.3015	13	11.62	4648.1	0.0813
7	7.55	3020.3	0.2003	14	11.64	4658.3	0.1955

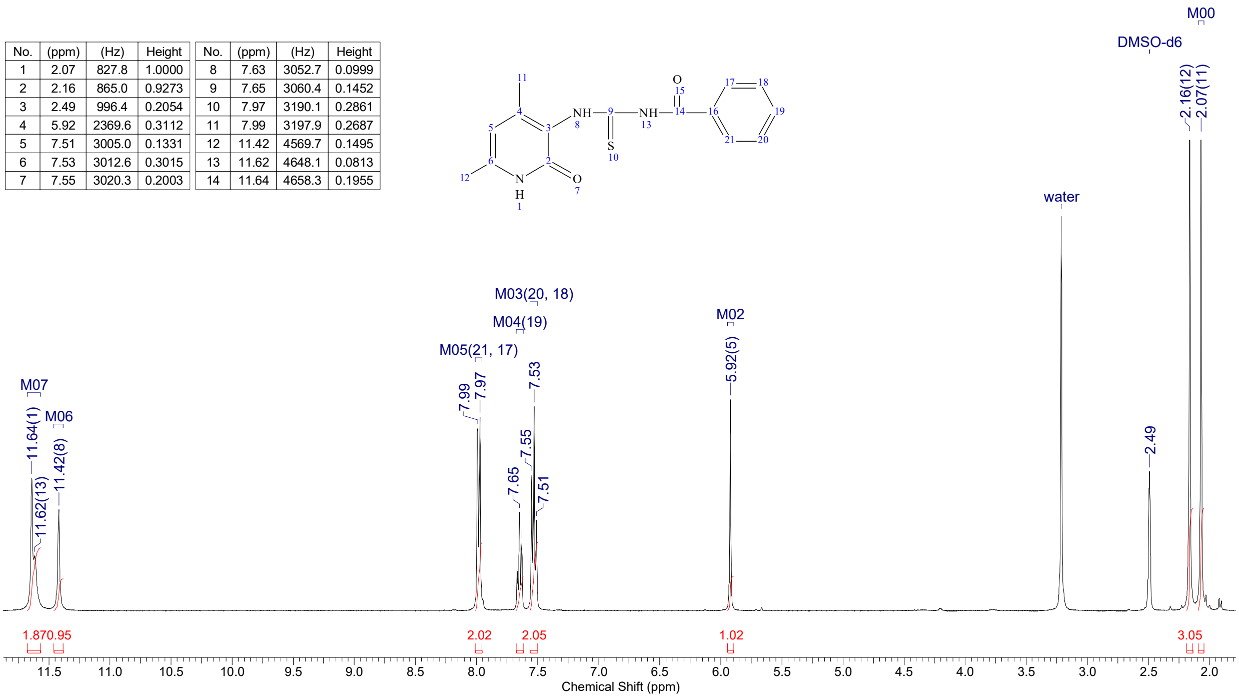

No.	(ppm)	(Hz)	Height	No.	(ppm)	(Hz)	Height
1	18.24	1835.4	-0.5162	7	133.10	13392.8	-0.1404
2	106.63	10729.0	-0.1450	8	143.07	14395.6	0.0747
3	123.41	12417.8	0.0937	9	147.60	14852.0	0.1034
4	128.45	12925.5	-0.4680	10	159.48	16047.0	0.1050
5	128.65	12945.3	-0.4777	11	168.28	16932.5	0.1004
6	132.15	13297.4	0.0986	12	180.93	18205.3	0.0952

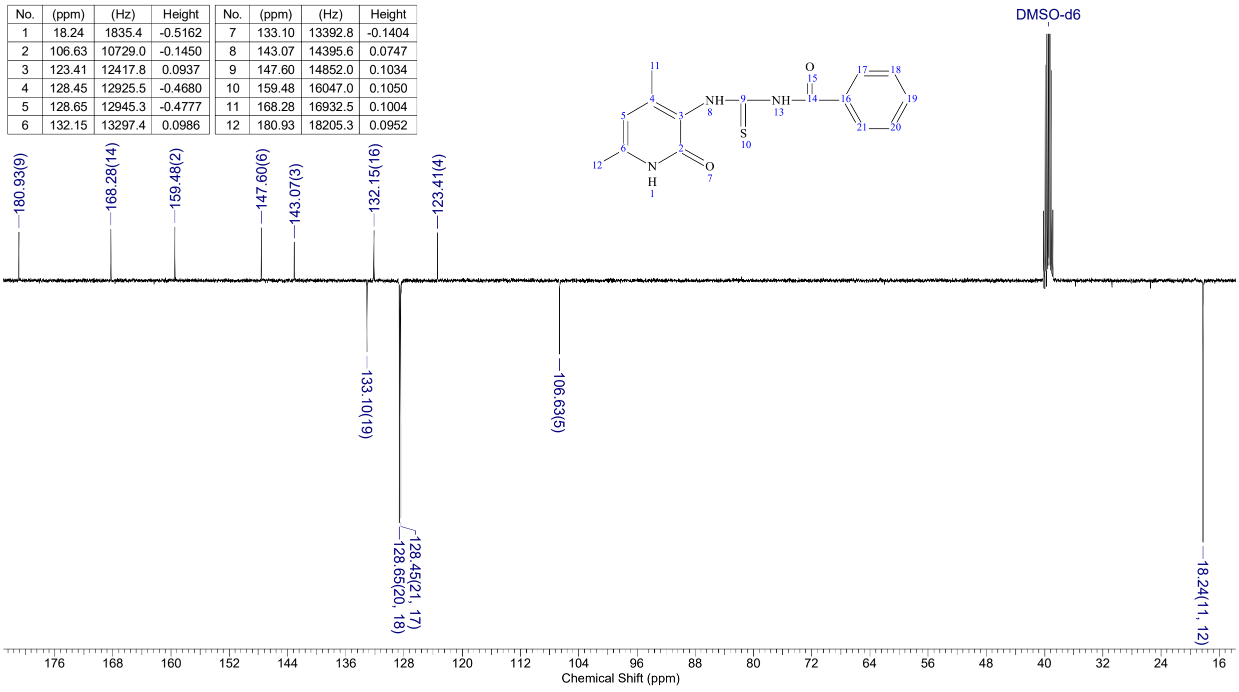

^{1}H (400 MHz, DMSO-d6) and ^{13}C (100 MHz, DMSO-d6) NMR Spectra of **11a**

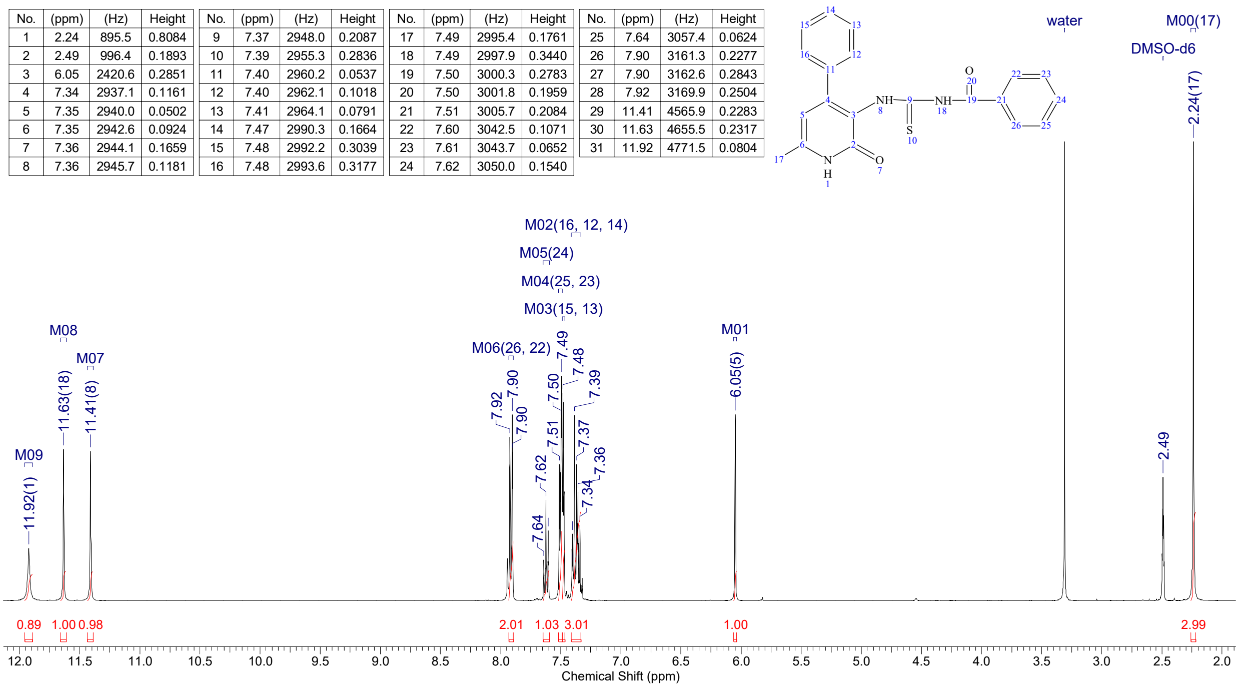

No.	(ppm)	(Hz)	Height
1	2.24	895.5	0.8084
2	2.49	996.4	0.1893
3	6.05	2420.6	0.2851
4	7.34	2937.1	0.1161
5	7.35	2940.0	0.0502
6	7.35	2942.6	0.0924
7	7.36	2944.1	0.1659
8	7.36	2945.7	0.1181
9	7.37	2948.0	0.2087
10	7.39	2955.3	0.2836
11	7.40	2960.2	0.0537
12	7.40	2962.1	0.1018
13	7.41	2964.1	0.0791
14	7.47	2990.3	0.1664
15	7.48	2992.2	0.3039
16	7.48	2993.6	0.3177
17	7.49	2995.4	0.1761
18	7.49	2997.9	0.3440
19	7.50	3000.3	0.2783
20	7.50	3001.8	0.1959
21	7.51	3005.7	0.2084
22	7.60	3042.5	0.1071
23	7.61	3043.7	0.0652
24	7.62	3050.0	0.1540
25	7.64	3057.4	0.0624
26	7.90	3161.3	0.2277
27	7.90	3162.6	0.2843
28	7.92	3169.9	0.2504
29	11.41	4565.9	0.2283
30	11.63	4655.5	0.2317
31	11.92	4771.5	0.0804

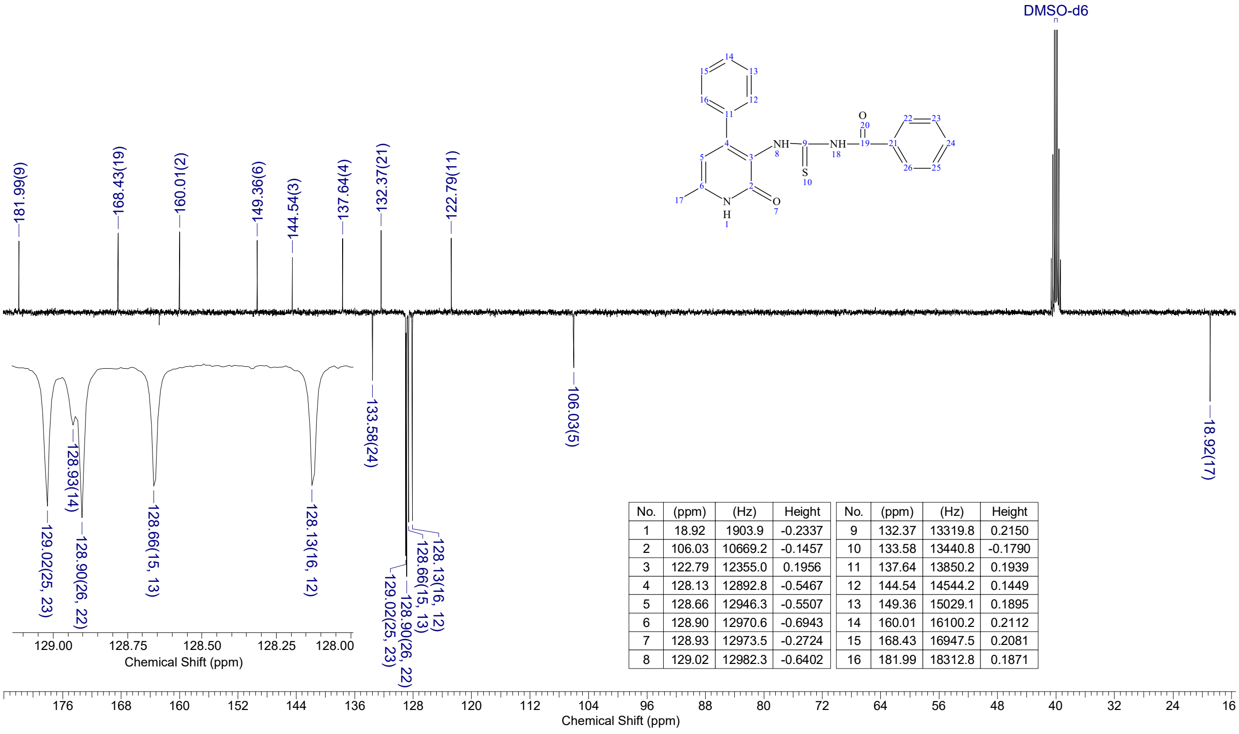

No.	(ppm)	(Hz)	Height
1	18.92	1903.9	-0.2337
2	106.03	10669.2	-0.1457
3	122.79	12355.0	0.1956
4	128.13	12892.8	-0.5467
5	128.66	12946.3	-0.5507
6	128.90	12970.6	-0.6943
7	128.93	12973.5	-0.2724
8	129.02	12982.3	-0.6402
9	132.37	13319.8	0.2150
10	133.58	13440.8	-0.1790
11	137.64	13850.2	0.1939
12	144.54	14544.2	0.1449
13	149.36	15029.1	0.1895
14	160.01	16100.2	0.2112
15	168.43	16947.5	0.2081
16	181.99	18312.8	0.1871

^{1}H (400 MHz, DMSO-d6) and ^{13}C (100 MHz, DMSO-d6) NMR Spectra of **11b**

No.	(ppm)	(Hz)	Height
1	2.23	1115.6	0.4505
2	3.34	1668.7	1.0000
3	6.50	3249.1	0.1447
4	7.14	3572.5	0.0632
5	7.15	3576.5	0.0759
6	7.15	3577.4	0.0774
7	7.16	3581.4	0.0663
8	7.53	3766.3	0.0703
9	7.55	3774.0	0.1480
10	7.56	3781.9	0.0959
11	7.65	3828.1	0.0478
12	7.67	3835.6	0.0762
13	7.69	3843.7	0.1102
14	7.69	3847.5	0.0838
15	7.72	3858.6	0.0825
16	7.73	3863.8	0.0813
17	8.00	4002.0	0.1392
18	8.02	4009.3	0.1297
19	11.76	5879.9	0.1338
20	11.81	5906.7	0.0435

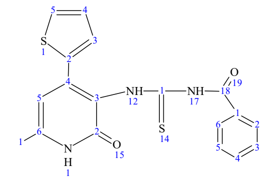

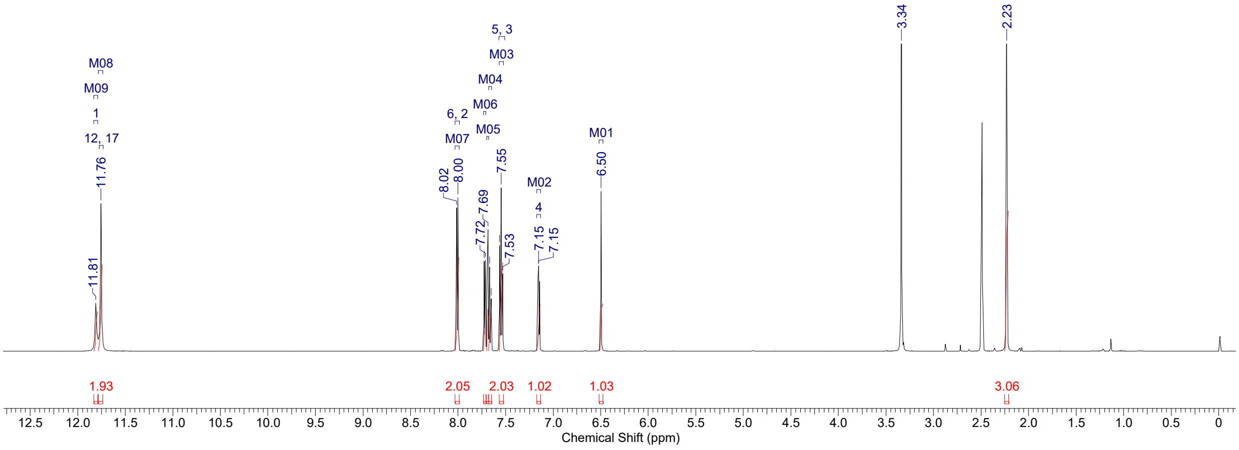

No.	(ppm)	(Hz)	Height
1	18.48	2324.3	0.0566
2	102.69	12913.7	0.0302
3	120.43	15144.6	0.0302
4	127.19	15995.3	0.0678
5	128.47	16156.1	0.1520
6	128.67	16181.3	0.1499
7	128.98	16220.9	0.0573
8	130.22	16375.7	0.0479
9	131.94	16592.0	0.0414
10	133.20	16751.0	0.0404
11	136.81	17205.4	0.0432
12	140.61	17683.4	0.0340
13	143.57	18055.3	0.0214
14	159.68	20080.8	0.0423
15	168.24	21158.0	0.0428
16	182.39	22936.9	0.0319

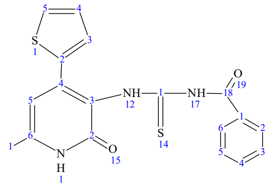

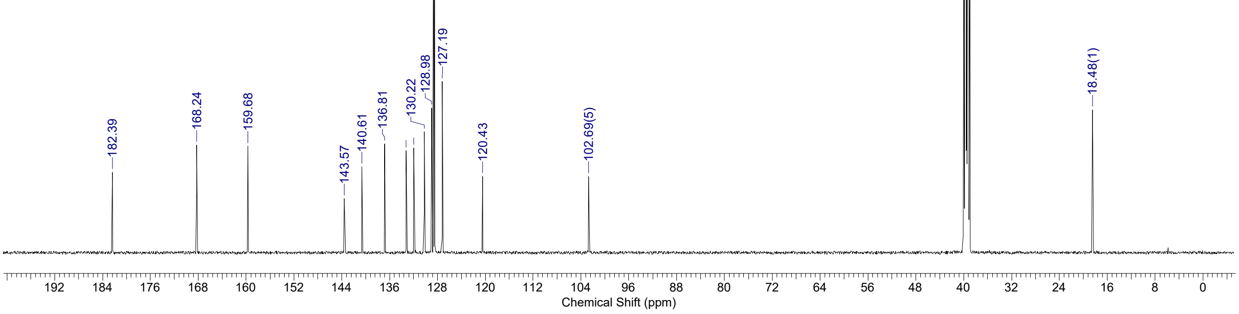

^{1}H (500 MHz, DMSO-d6) and ^{13}C (125 MHz, DMSO-d6) NMR Spectra of **11c**

Copies of MS Spectra of Products

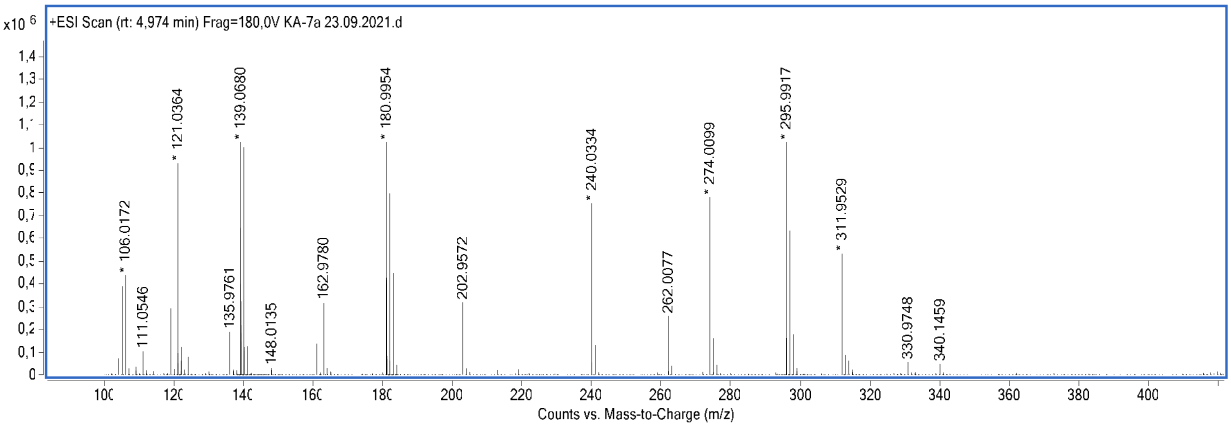

Mass spectrum (LC/Q-TOF) of (**9a**)

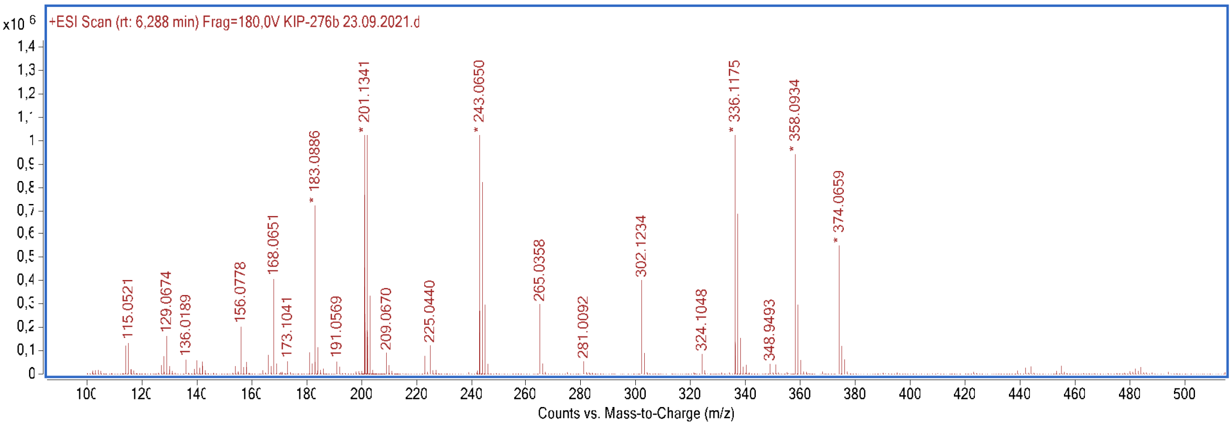

Mass spectrum (LC/Q-TOF) of (**9b**)

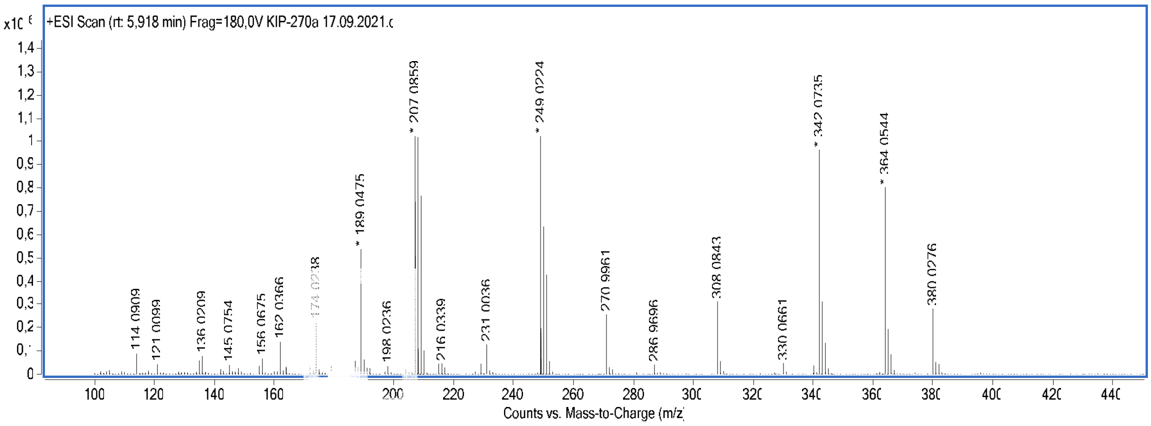

Mass spectrum (LC/Q-TOF) of (**9c**)

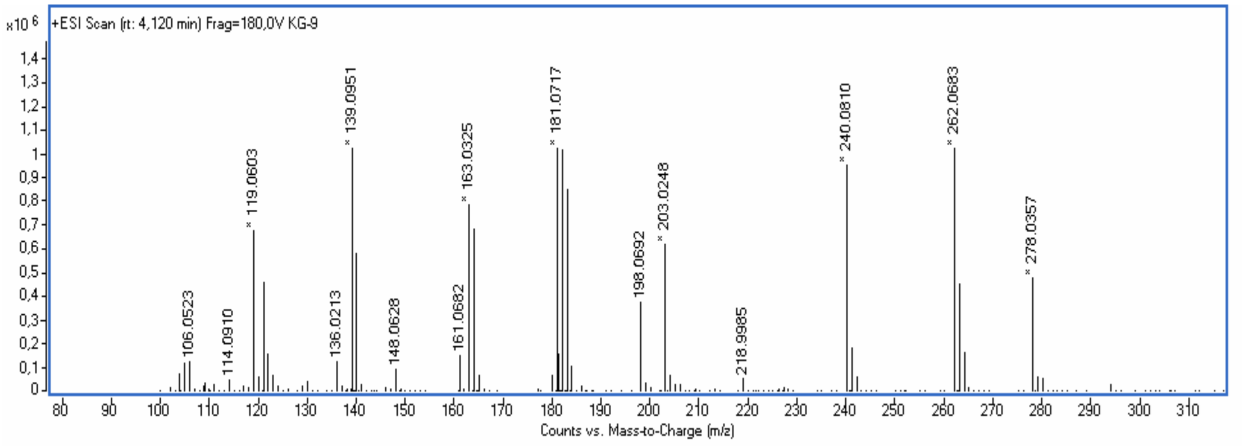

Mass spectrum (LC/Q-TOF) of (**10a**)

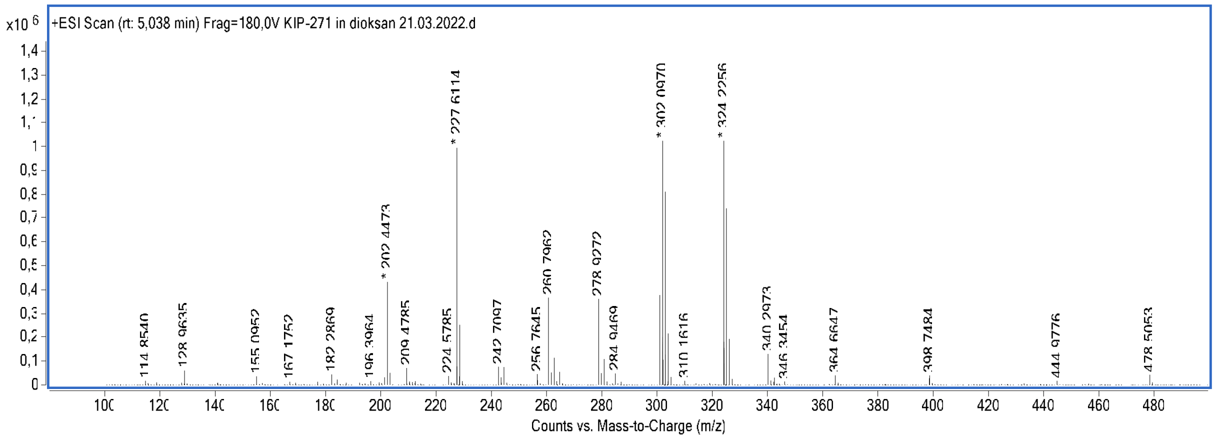

Mass spectrum (LC/Q-TOF) of (**10b**)

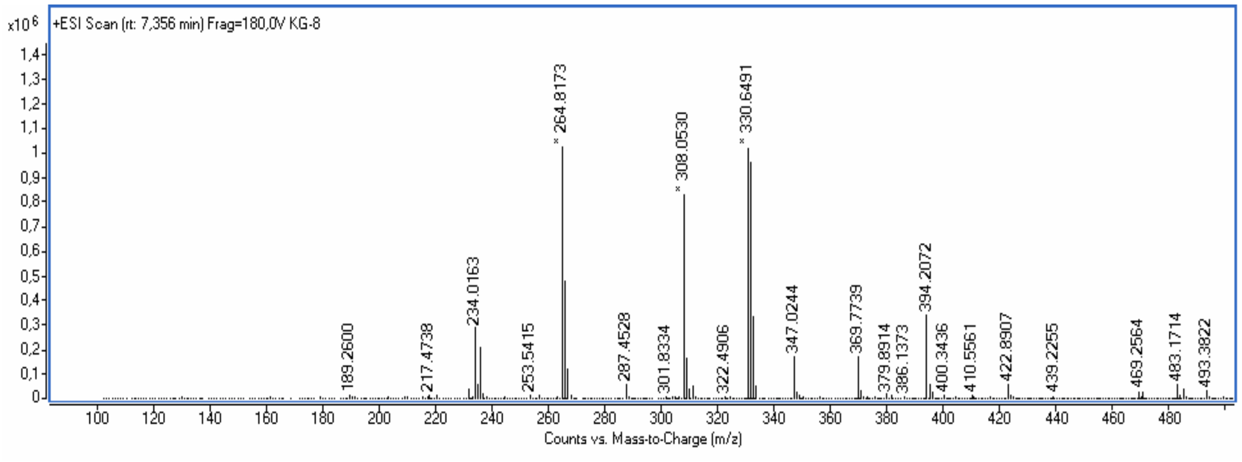

Mass spectrum (LC/Q-TOF) of (**10c**)

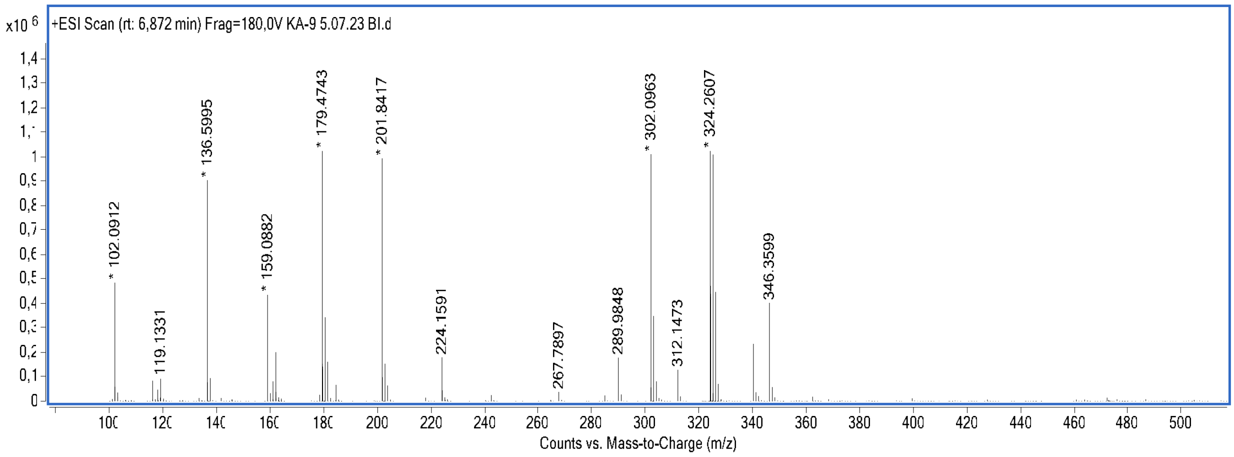

Mass spectrum (LC/Q-TOF) of (**11a**)

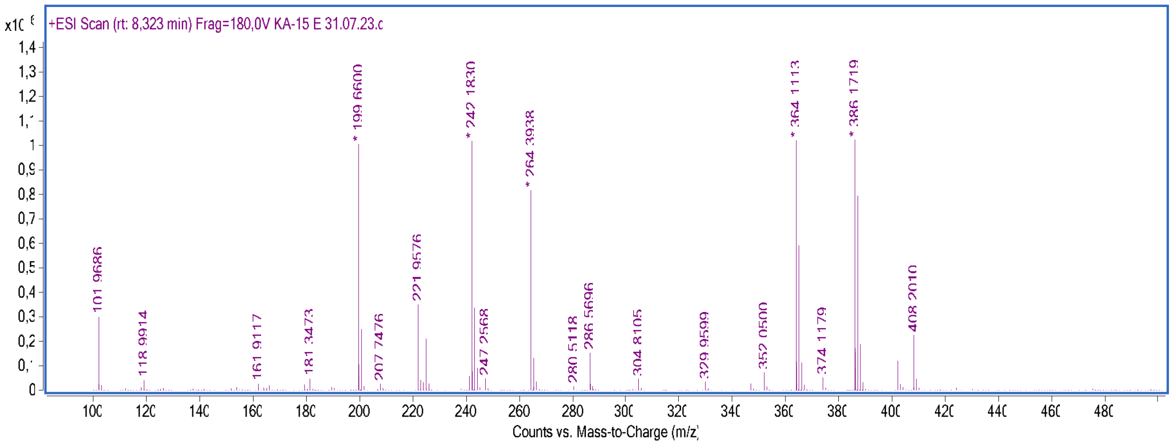

Mass spectrum (LC/Q-TOF) of (**11b**)

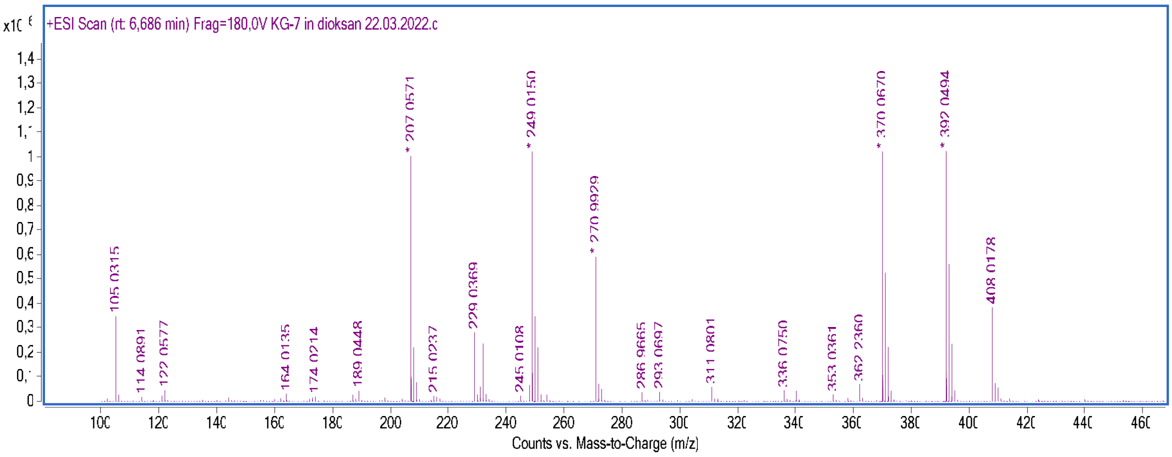

Mass spectrum (LC/Q-TOF) of (**11c**)

Table S1. Complexes between synthesized derivatives 8-11(a-c) and active sites of proteins (PDB: 3A4A, 5NN8)

	3A4A		5NN8	
	3D docking model	2D docking model	3D docking model	2D docking model
8a	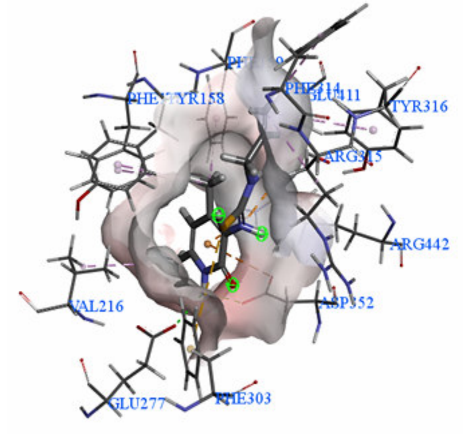	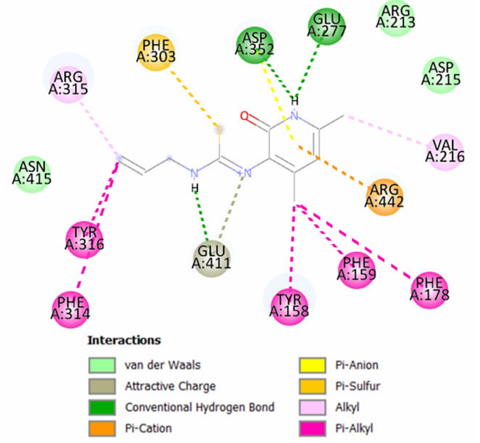	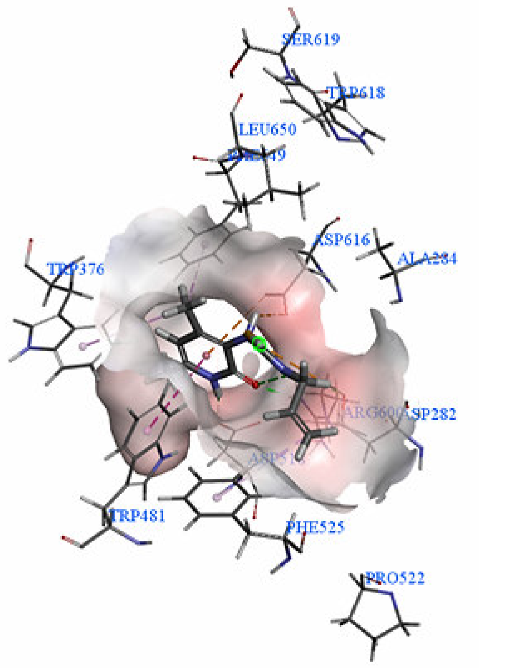	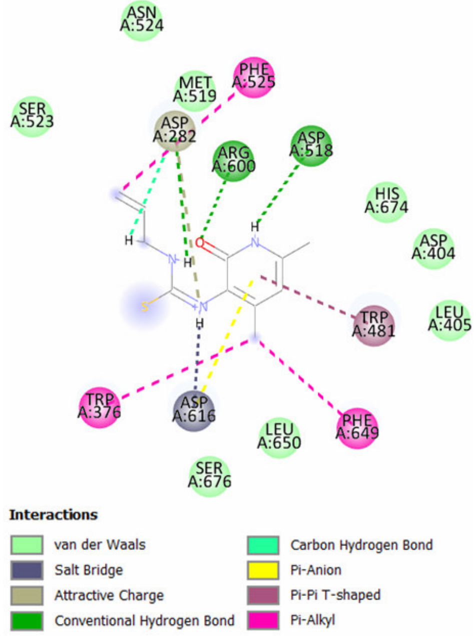

8b

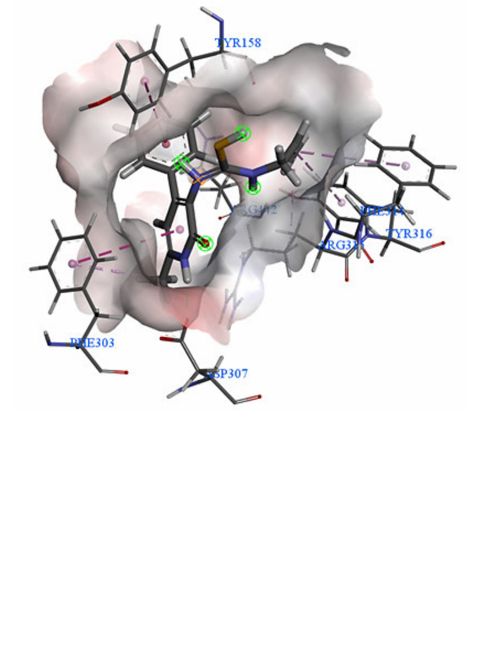
TYR158
TYR316
PHE303

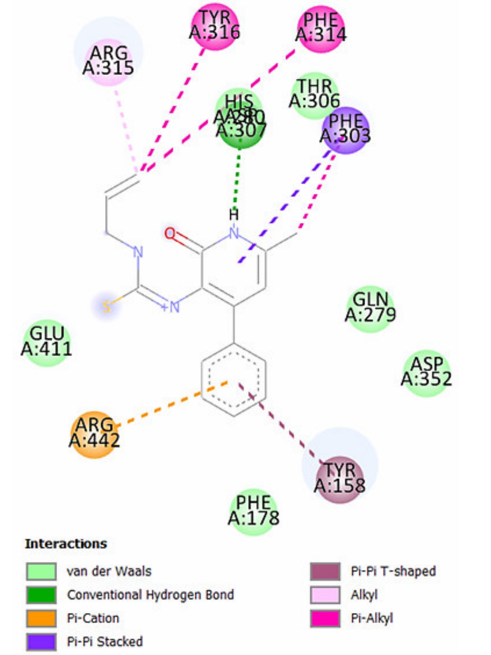
ARG A:315
TYR A:316
PHE A:314
THR A:306
PHE A:303
GLU A:411
GLN A:279
ASP A:352
ARG A:442
TYR A:158
PHE A:178
Interactions
van der Waals
Conventional Hydrogen Bond
Pi-Cation
Pi-Pi Stacked
Pi-Pi T-shaped
Alkyl
Pi-Alkyl

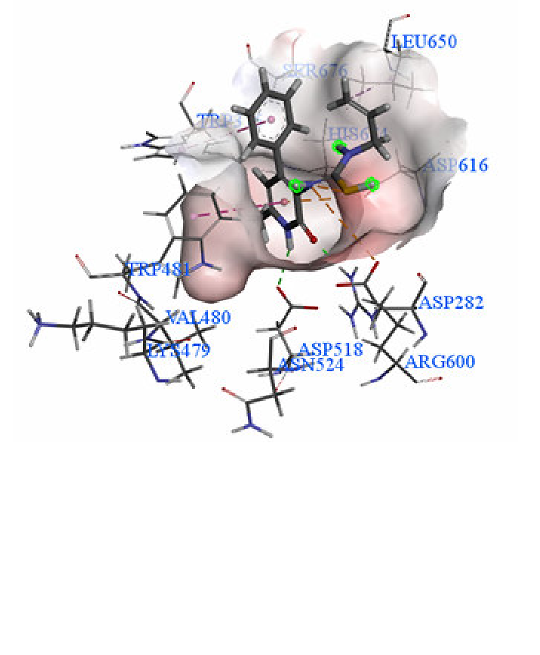
LEU650
ASP616
TRP481
VAL480
LYS479
ASP518
ASN524
ASP282
ARG600

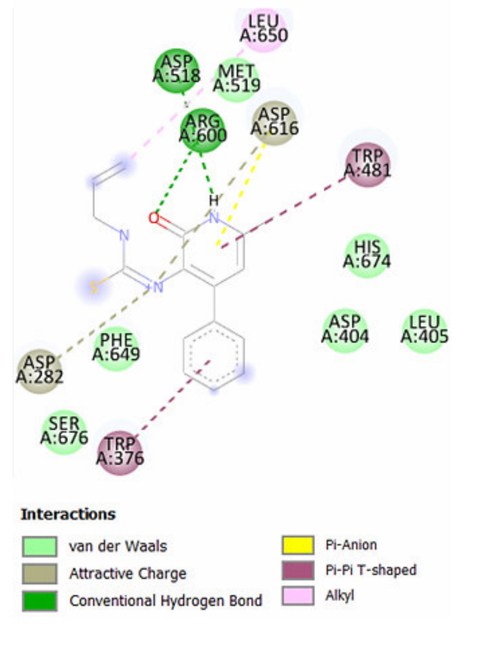
LEU A:650
ASP A:518
MET A:519
ARG A:600
ASP A:616
TRP A:481
HIS A:674
ASP A:404
LEU A:405
ASP A:282
PHE A:649
SER A:676
TRP A:376
Interactions
van der Waals
Attractive Charge
Conventional Hydrogen Bond
Pi-Anion
Pi-Pi T-shaped
Alkyl

8c

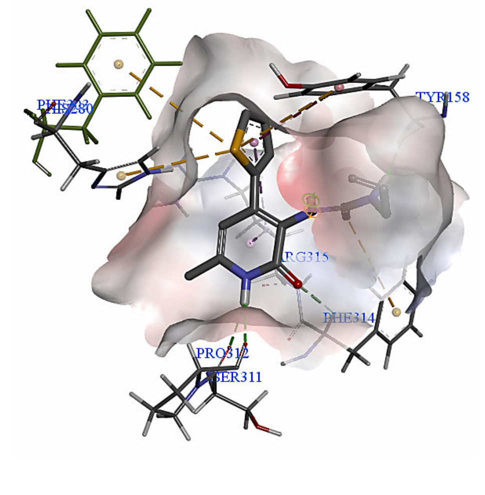
TYR158
PHE314
PRO312
SER311

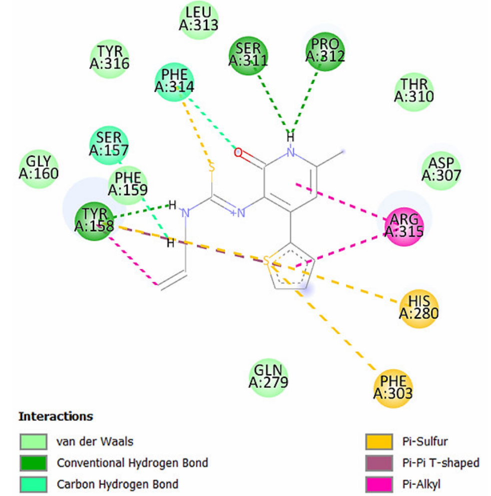
LEU A:313
TYR A:316
SER A:311
PRO A:312
PHE A:314
THR A:310
SER A:157
GLY A:160
PHE A:159
ASP A:307
TYR A:158
ARG A:315
HIS A:280
GLN A:279
PHE A:303
Interactions
van der Waals
Conventional Hydrogen Bond
Carbon Hydrogen Bond
Pi-Sulfur
Pi-Pi T-shaped
Pi-Alkyl

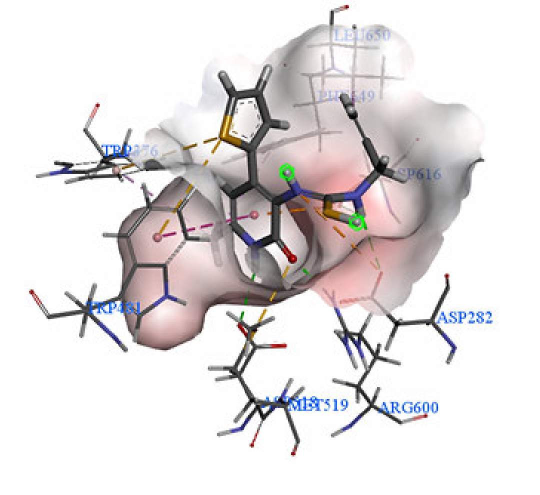
ASP282
ARG600

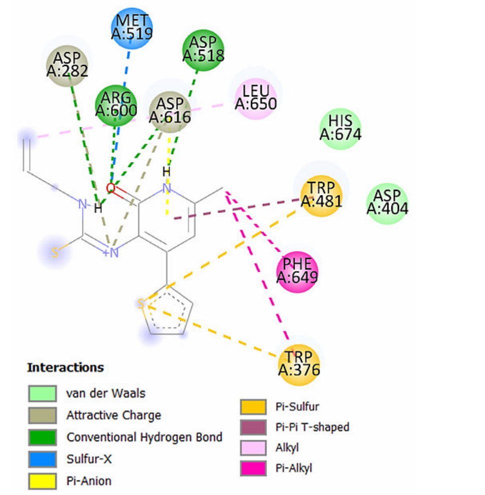
MET A:519
ASP A:282
ASP A:518
ARG A:600
ASP A:616
LEU A:650
HIS A:674
TRP A:481
ASP A:404
PHE A:649
TRP A:376
Interactions
van der Waals
Attractive Charge
Conventional Hydrogen Bond
Sulfur-X
Pi-Anion
Pi-Sulfur
Pi-Pi T-shaped
Alkyl
Pi-Alkyl

9a

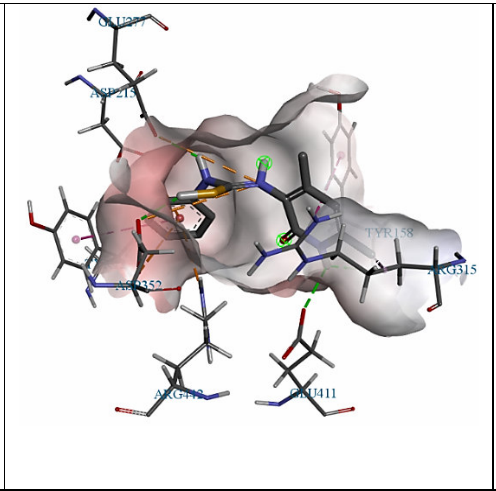
GLU277
ASP215
TYR158
ASP352
ARG315
ARG442
GLU411

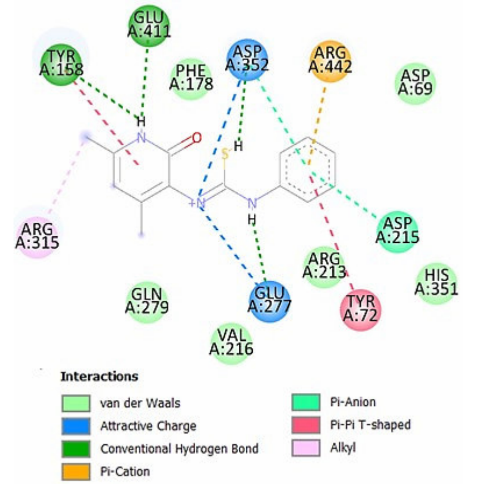
GLU A:411
TYR A:158
PHE A:178
ASP A:352
ARG A:442
ASP A:69
ARG A:315
ASP A:215
ARG A:213
HIS A:351
GLN A:279
GLU A:277
TYR A:72
VAL A:216
Interactions
van der Waals
Attractive Charge
Conventional Hydrogen Bond
Pi-Cation
Pi-Anion
Pi-Pi T-shaped
Alkyl

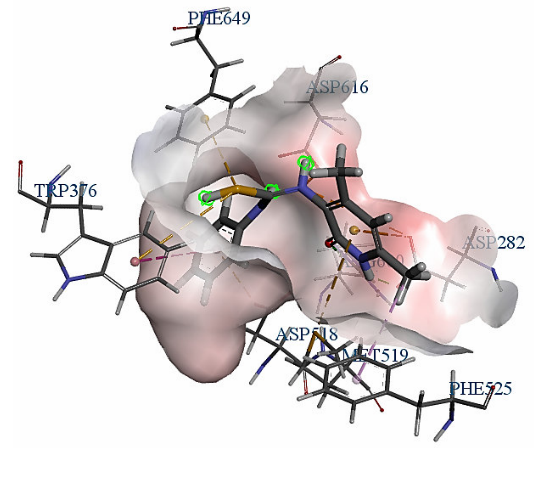
PHE649
ASP616
TRP376
ASP282
ASP518
MET519
PHE525

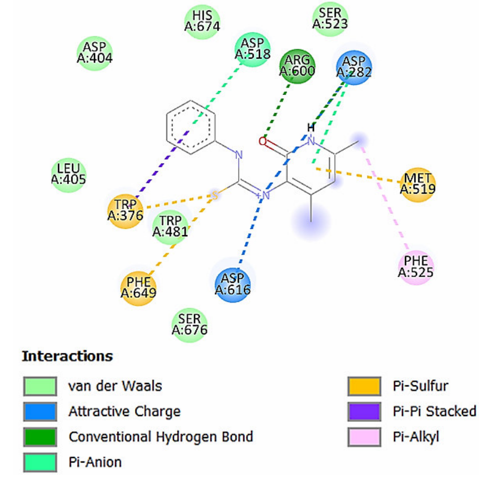
HIS A:674
SER A:523
ASP A:404
ASP A:518
ARG A:600
ASP A:282
LEU A:405
MET A:519
TRP A:376
TRP A:481
PHE A:525
PHE A:649
ASP A:616
SER A:676
Interactions
van der Waals
Attractive Charge
Conventional Hydrogen Bond
Pi-Anion
Pi-Sulfur
Pi-Pi Stacked
Pi-Alkyl

9b

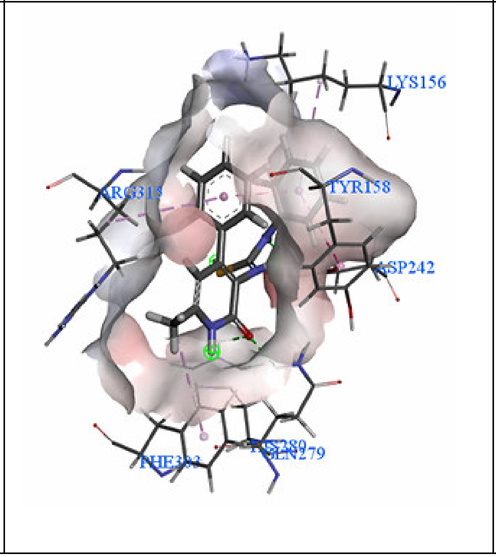
LYS156
ARG315
TYR158
ASP242
GLN279

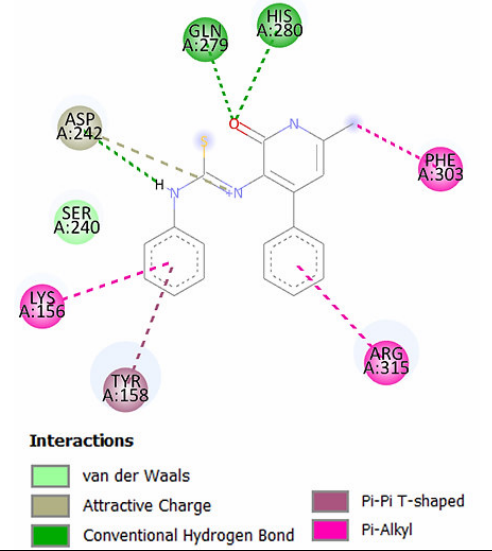
GLN A:279
HIS A:280
ASP A:242
PHE A:303
SER A:240
LYS A:156
TYR A:158
ARG A:315
Interactions
van der Waals
Attractive Charge
Conventional Hydrogen Bond
Pi-Pi T-shaped
Pi-Alkyl

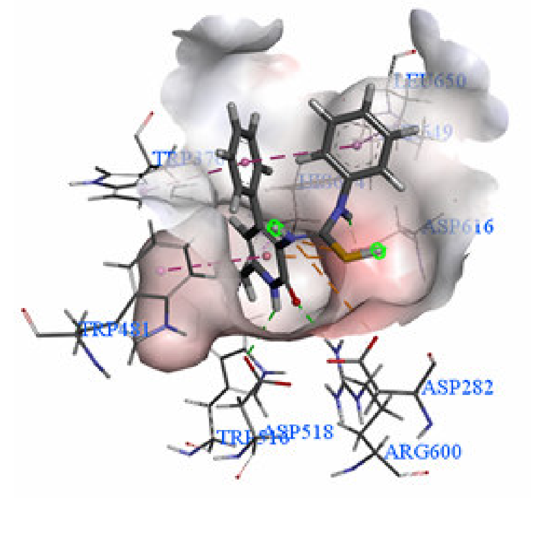
LEU650
ASP616
TRP481
ASP282
ASP518
ARG600

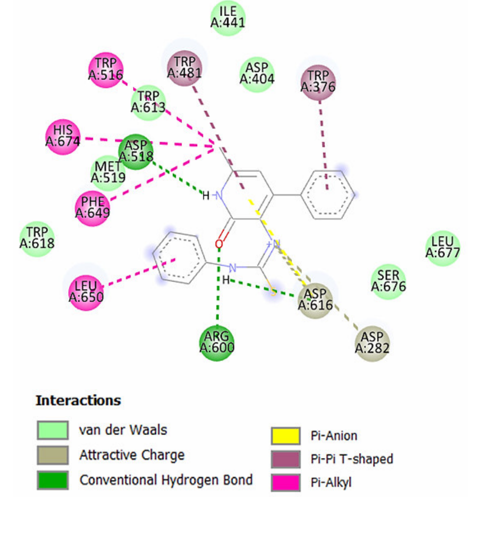
ILE A:441
TRP A:516
TRP A:481
ASP A:404
TRP A:376
TRP A:613
HIS A:674
ASP A:518
MET A:519
PHE A:649
TRP A:618
LEU A:677
SER A:676
LEU A:650
ASP A:616
ARG A:600
ASP A:282
Interactions
van der Waals
Attractive Charge
Conventional Hydrogen Bond
Pi-Anion
Pi-Pi T-shaped
Pi-Alkyl

9c

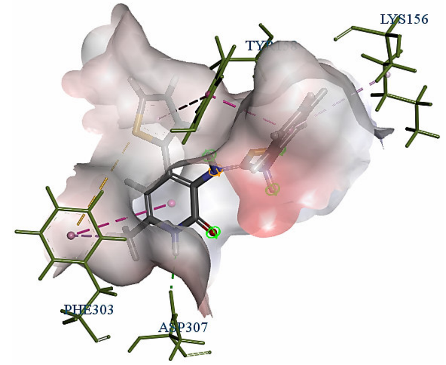
LYS156
PHE303
ASP307

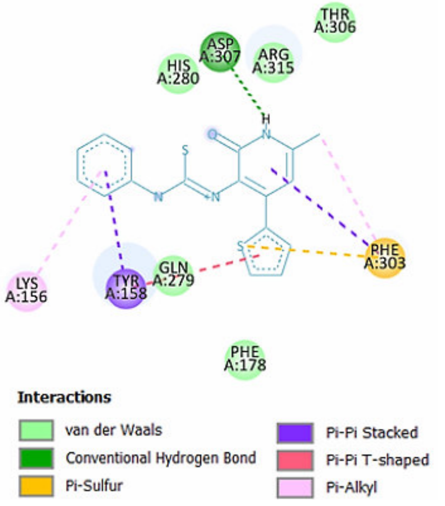
THR A:306
ASP A:307
HIS A:280
ARG A:315
LYS A:156
TYR A:158
GLN A:279
PHE A:303
PHE A:178
Interactions
van der Waals
Conventional Hydrogen Bond
Pi-Sulfur
Pi-Pi Stacked
Pi-Pi T-shaped
Pi-Alkyl

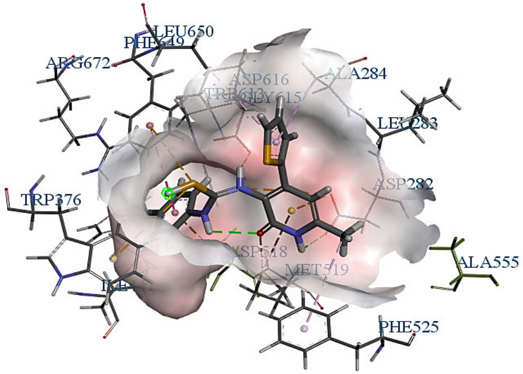
LEU650
PHE649
ARG672
ASP616
ALA284
LEU283
TRP376
ASP282
ALA555
MET519
PHE525

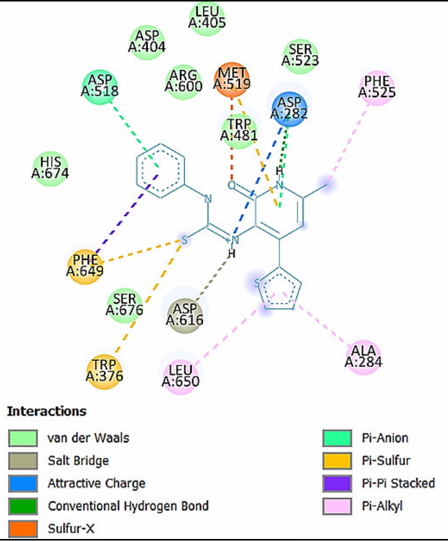
LEU A:405
ASP A:404
SER A:523
ASP A:518
ARG A:600
MET A:519
PHE A:525
ASP A:282
TRP A:481
HIS A:674
PHE A:649
SER A:676
ASP A:616
TRP A:376
LEU A:650
ALA A:284
Interactions
van der Waals
Salt Bridge
Attractive Charge
Conventional Hydrogen Bond
Sulfur-X
Pi-Anion
Pi-Sulfur
Pi-Pi Stacked
Pi-Alkyl

10a

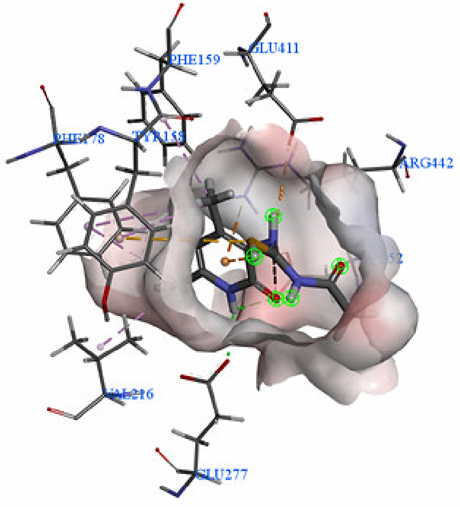
PHE159
GLU411
PHE178
TYR158
ARG442
VAL216
GLU277

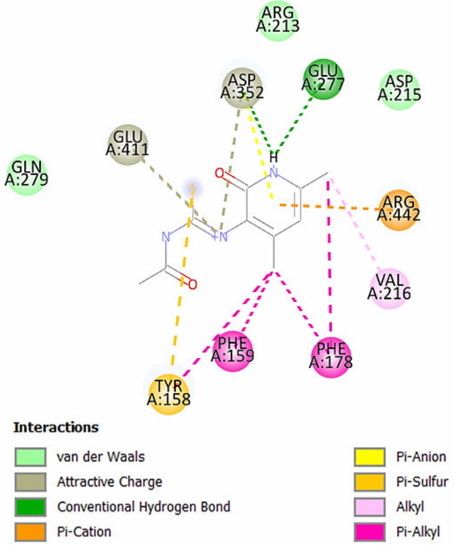
ARG A:213
ASP A:352
GLU A:277
ASP A:215
GLU A:411
GLN A:279
ARG A:442
VAL A:216
PHE A:159
PHE A:178
TYR A:158
Interactions
van der Waals
Attractive Charge
Conventional Hydrogen Bond
Pi-Cation
Pi-Anion
Pi-Sulfur
Alkyl
Pi-Alkyl

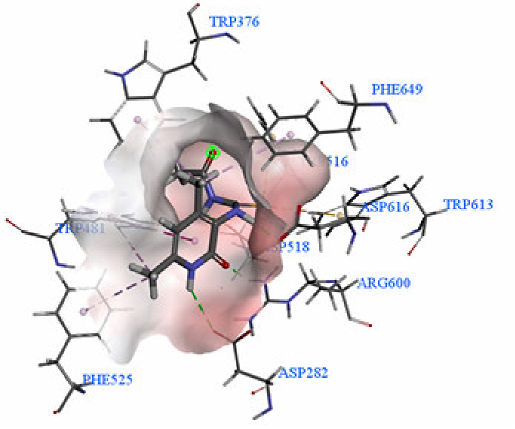
TRP376
PHE649
ASP616
TRP613
ARG600
PHE525
ASP282

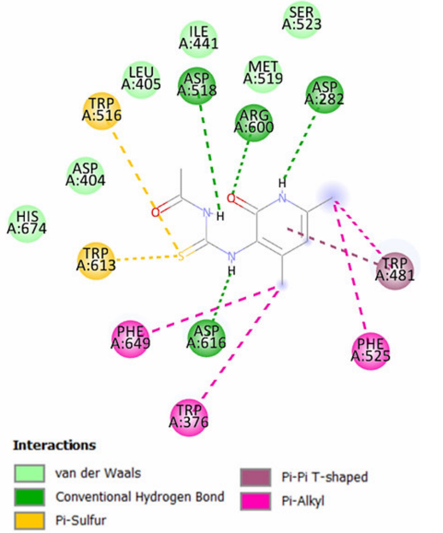
SER A:523
ILE A:441
LEU A:405
ASP A:518
MET A:519
TRP A:516
ARG A:600
ASP A:282
ASP A:404
HIS A:674
TRP A:613
TRP A:481
PHE A:649
ASP A:616
PHE A:525
TRP A:376
Interactions
van der Waals
Conventional Hydrogen Bond
Pi-Sulfur
Pi-Pi T-shaped
Pi-Alkyl

10b

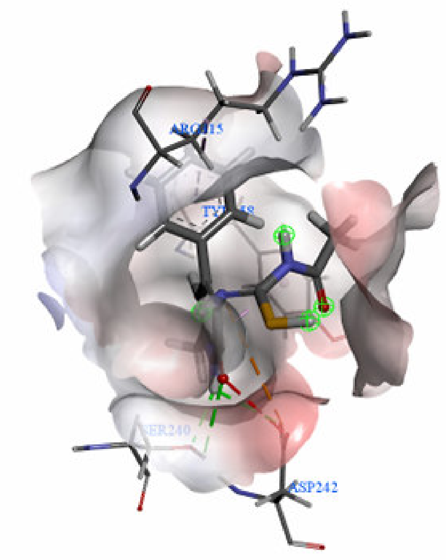

ARG315
SER240
ASP242

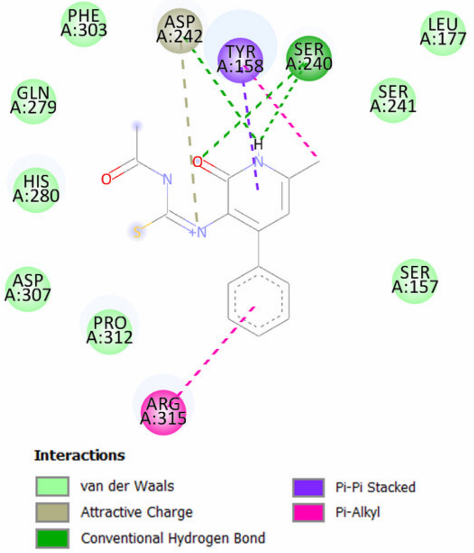

PHE A:303
ASP A:242
TYR A:158
SER A:240
LEU A:177
GLN A:279
SER A:241
HIS A:280
ASP A:307
SER A:157
PRO A:312
ARG A:315
Interactions
van der Waals
Attractive Charge
Conventional Hydrogen Bond
Pi-Pi Stacked
Pi-Alkyl

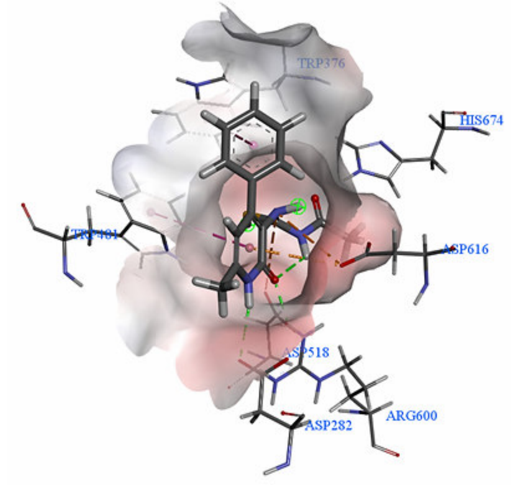

TRP376
HIS674
TRP481
ASP616
ASP518
ASP282
ARG600

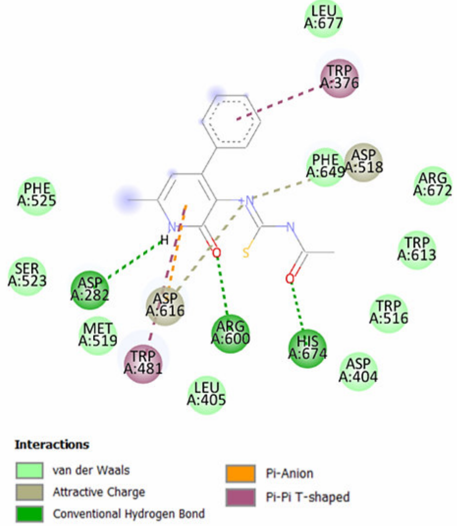

LEU A:677
TRP A:376
PHE A:649
ASP A:518
PHE A:525
ARG A:672
TRP A:613
SER A:523
ASP A:282
ASP A:616
TRP A:516
MET A:519
ARG A:600
HIS A:674
TRP A:481
ASP A:404
LEU A:405
Interactions
van der Waals
Attractive Charge
Conventional Hydrogen Bond
Pi-Anion
Pi-Pi T-shaped

10c

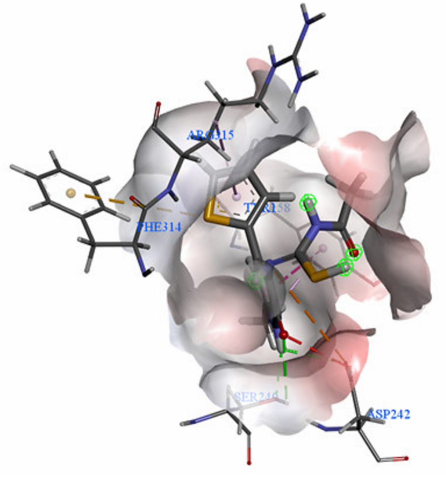

PHE314
SER240
ASP242

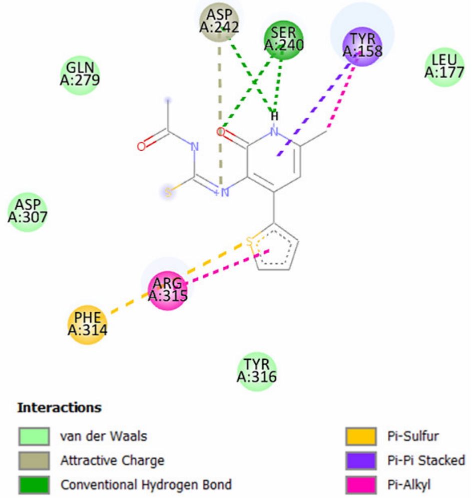

ASP A:242
SER A:240
TYR A:158
GLN A:279
LEU A:177
ASP A:307
ARG A:315
PHE A:314
TYR A:316
Interactions
van der Waals
Attractive Charge
Conventional Hydrogen Bond
Pi-Sulfur
Pi-Pi Stacked
Pi-Alkyl

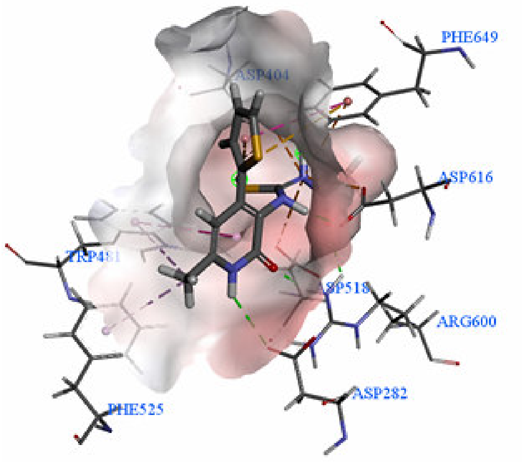

PHE649
ASP404
ASP616
TRP481
ASP518
ARG600
PHE525
ASP282

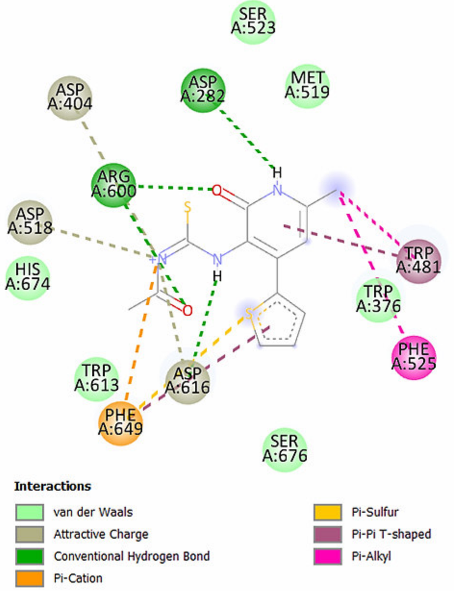

SER A:523
ASP A:404
ASP A:282
MET A:519
ARG A:600
ASP A:518
HIS A:674
TRP A:481
TRP A:376
PHE A:525
TRP A:613
ASP A:616
PHE A:649
SER A:676
Interactions
van der Waals
Attractive Charge
Conventional Hydrogen Bond
Pi-Cation
Pi-Sulfur
Pi-Pi T-shaped
Pi-Alkyl

<table>
<tr>
<td>11 a</td>
<td>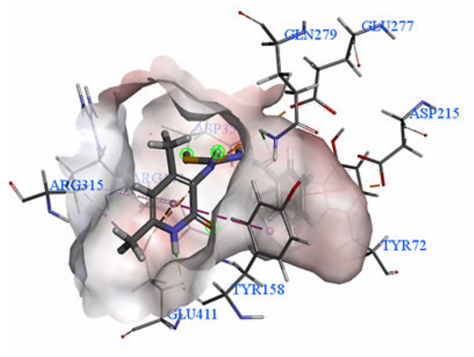
</td>
<td>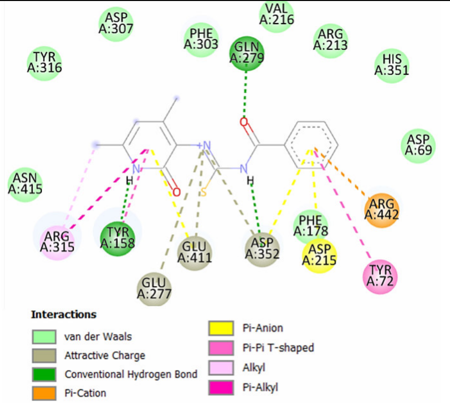
</td>
<td>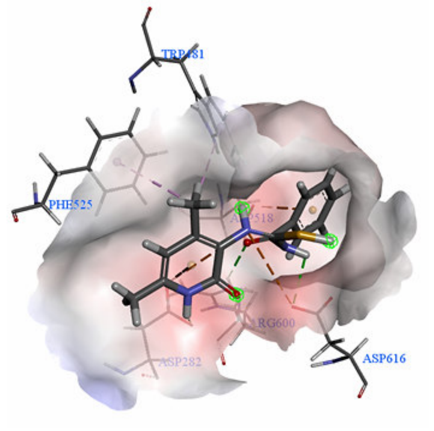
</td>
<td>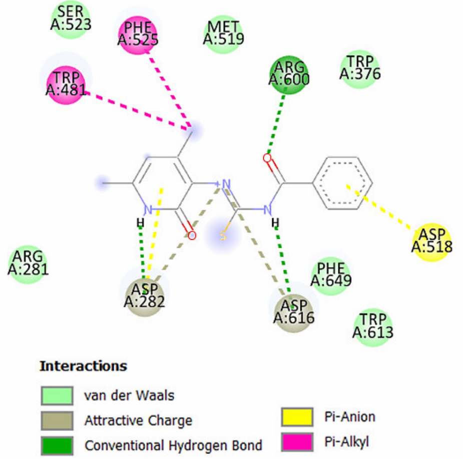
</td>
</tr>
<tr>
<td>11b</td>
<td>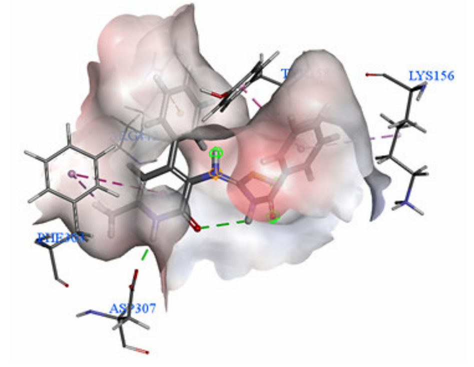
</td>
<td>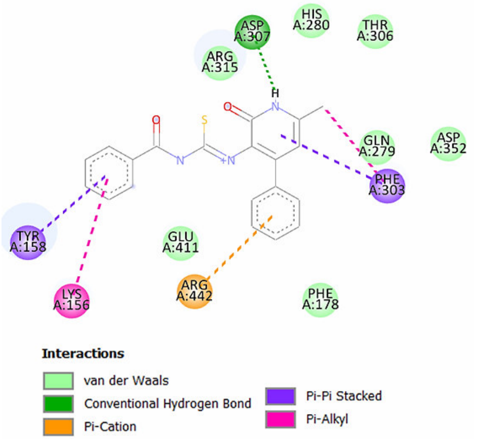
</td>
<td>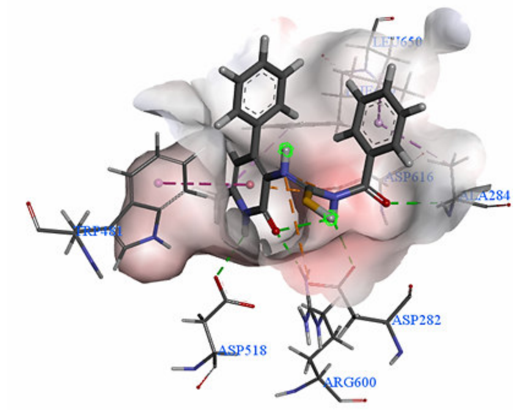
</td>
<td>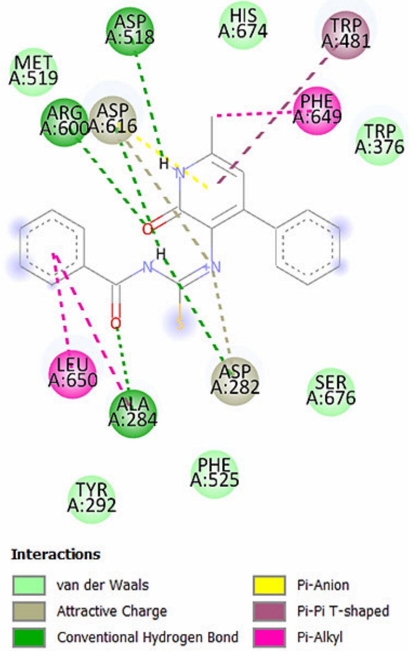
</td>
</tr>
</table>

11c	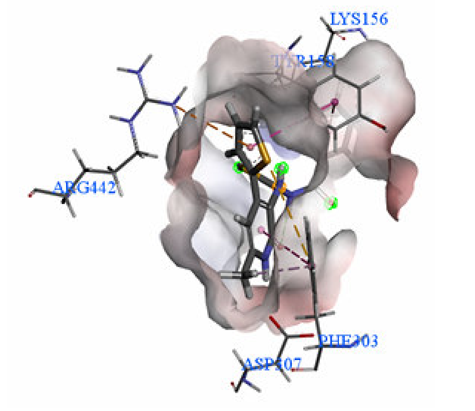 LYS156<br>ARG442<br>PHE303<br>ASP307	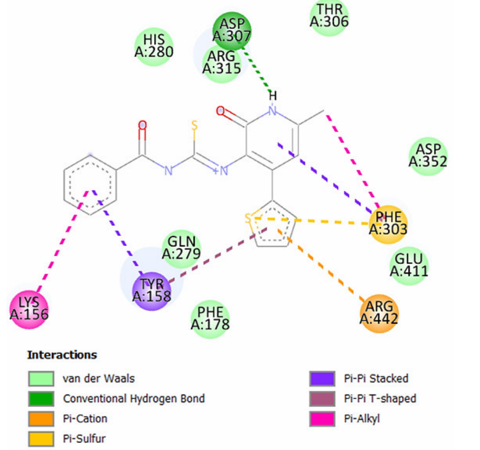 HIS A:280<br>ASP A:307<br>ARG A:315<br>THR A:306<br>ASP A:352<br>PHE A:303<br>GLN A:279<br>GLU A:411<br>LYS A:156<br>TYR A:158<br>PHE A:178<br>ARG A:442<br>Interactions<br>van der Waals<br>Conventional Hydrogen Bond<br>Pi-Cation<br>Pi-Sulfur<br>Pi-Pi Stacked<br>Pi-Pi T-shaped<br>Pi-Alkyl	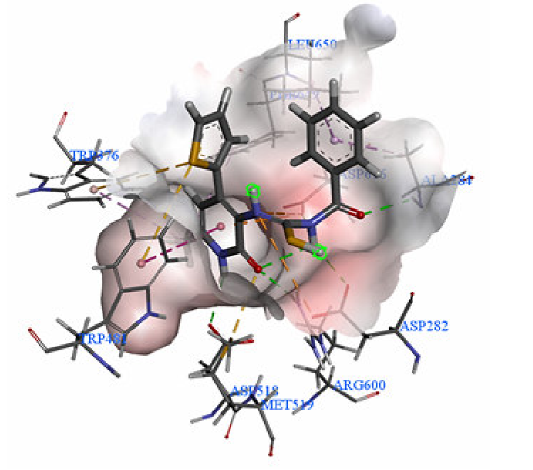 LEU650<br>TRP376<br>ASP282<br>ASP518<br>MET519<br>ARG600	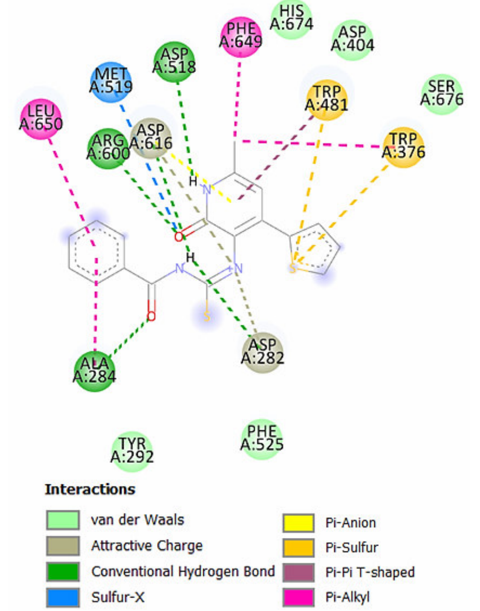 HIS A:674<br>PHE A:649<br>ASP A:404<br>ASP A:518<br>MET A:519<br>TRP A:481<br>SER A:676<br>LEU A:650<br>ARG A:600<br>ASP A:616<br>TRP A:376<br>ALA A:284<br>ASP A:282<br>TYR A:292<br>PHE A:525<br>Interactions<br>van der Waals<br>Attractive Charge<br>Conventional Hydrogen Bond<br>Sulfur-X<br>Pi-Anion<br>Pi-Sulfur<br>Pi-Pi T-shaped<br>Pi-Alkyl

Table S2. Basic amino acid interactions and H-bonds

Compound	Receptor	H-bond	Residual Amino acid Interactions	
			Pi-Sulfur/ Pi-Anion/Pi-Pi Stacked/ Pi-Pi T-shaped/Pi-Alkyl/Amide-Pi Stacked/Pis interactions	Van-der Walls interactions
8a	3a4a	ASP352, GLU277, GLU411	TYR316, PHE314, GLU411, TYR158, PHE159, PHE178, ARG442, VAL216, ASP352, PHE303, ARG315	ASN415, ARG213, ASP215
8b		ASP307	ARG442, TYR158, ARG315, TYR316, PHE314, PHE303	GLU411, PHE178, ASP352, GLN279, HIS280, THR306
8c		SER157, PHE314, SER311, PRO312, TYR158	TYR158, PHE314, ARG315, HIS280, PHE303	GLY160, PHE159, GLN279, THR310, ASP307, TYR316, LEU313
9a		TYR158, GLU411, ASP352, GLU277	ARG315, GLU277, TYR72, ASP215, ARG442, ASP352, TYR158	PHE178, GLN279, VAL216, HIS351, ARG213, ASP69
9b		GLN279, HIS280, ASP242	ASP242, PHE303, ARG315, TYR158, LYS156	SER240

9c		ASP307	LYS156, TYR158, PHE303	HIS280, GLN279, PHE178, ARG315, THR306
10a		ASP352, GLU277	GLU411, ASP352, ARG442, VAL216, PHE178, PHE159, TYR158	GLN279, ARG213, ASP215
10b		ASP242, SER240	ASP242, TYR158, SER240, ARG315	PRO312, ASP307, HIS280, GLN279, PHE303, LEU177, SER241, SER157
10c		ASP242, SER240	ASP242, TYR158, ARG315, PHE314	ASP307, GLN279, LEU177, TYR316
11a		TYR158, GLN279, ASP352	ARG315, TYR158, GLU277, GLU411, ASP352, ASP215, TYR72, ARG442	ASN415, TYR316, ASP307, PHE303, VAL216, ARG213, HIS351, ASP69, PHE178
11b		ASP307	TYR158, LYS156, ARG442, PHE303	GLU411, PHE178, GLN279, ARG315, HIS280, THR306
11c		ASP307	LYS156, TYR158, ARG442, PHE303	GLN279, PHE178, GLU411, ASP352, THR306, ARG315, HIS280
8a	5NN8	ASP282, ARG600, ASP518	PHE525, ASP282, ASP616, TRP376, PHE649, TRP481	SER523, ASN524, MET519, HIS674, ASP404, LEU405, LEU650, SER676
8b		ASP518, ARG600	LEU650, ASP616, TRP481,	PHE649, SER676, MET519,

			TRP376, ASP282	HIS674, ASP404, LEU405
8c		ASP282, ARG600, ASP518	MET519, ASP282, ASP616, LEU650, TRP481, PHE649, TRP376	HIS674, ASP404
9a		ARG600, ASP282	TRP376, PHE649, ASP616, MET519, PHE525, ASP282, ASP518	ASP404, LEU405, TRP481, SER676, SER523, HIS674
9b		ASP518, ARG600, ASP616	TRP481, TRP516, HIS674, PHE649, LEU650, ASP616, ASP282, TRP376	ILE441, ASP404, TRP613, MET519, TRP618, SER676, LEU677
9c		ASP282	ASP518, PHE649, TRP376, ASP616, LEU650, ALA284, PHE525, ASP282, MET519	HIS674, SER676, TRP481, SER523, LEU405, ARG600, ASP404
10a		ASP616, ASP282, ARG600, ASP518	TRP516, TRP613, PHE649, TRP376, PHE525, TRP481	HIS674, ASP404, LEU405, ILE441, MET519, SER523
10b		ASP282, ARG600, HIS674	ASP616, TRP481, ASP518, TRP376	PHE525, SER523, MET519, LEU405, ASP404, TRP516, TRP613, ARG672, PHE649, LEU677
10c		ARG600, ASP282	ASP404, ASP518, PHE649, ASP616, PHE525, TRP481	HIS674, TRP613, SER676, TRP376, MET519, SER523
11a		ARG600, ASP282,	ASP282, ASP616, PHE525,	ARG281, SER523, MET519,

		ASP616	TRP481, ASP518	TRP376, PHE649, TRP613
11b		ALA284, ASP282, ARG600, ASP518, ASP616	ASP518, ASP616, LEU650, ALA284, PHE649, TRP481	MET519, TRP292, PHE525, SER676, TRP376, HIS674
11c		ALA284, ARG600, ASP518, ASP616, ASP282	ASP616, ASP282, MET519, LEU650, ALA284, PHE649, TRP481, TRP376	TYR292, PHE525, HIS674, ASP404, SER676
